# Single-cell transcriptomics and a boundary wound model reveal temporal decoupling and microenvironmental heterogeneity in radiation-induced fibrosis

**DOI:** 10.3389/fonc.2026.1871514

**Published:** 2026-08-20

**Authors:** Bingyue Cao, Pan Liu, Yujia Tan, Yongchang Wei

**Affiliations:** 1 Department of Radiation and Medical Oncology, Zhongnan Hospital of Wuhan University, Wuhan University, Wuhan, China; 2 Hubei Key Laboratory of Tumor Biological Behaviors, Zhongnan Hospital of Wuhan University, Wuhan University, Wuhan, China; 3 Hubei Cancer Clinical Study Center, Zhongnan Hospital of Wuhan University, Wuhan University, Wuhan, China; 4 Department of Clinical Laboratory, Renmin Hospital of Wuhan University, Wuhan, China

**Keywords:** fibrosis mechanism, mouse model, radiation injury, radiation-induced fibrosis, single-cell RNA-seq, SPP1/osteopontin, TGF-β signaling, wound healing

## Abstract

**Background:**

Radiotherapy is a key treatment method for cancer, but high-dose treatment can lead to damage to normal tissues and chronic fibrosis. There is a lack of systematic temporal analysis of the dynamic progression from early damage signals to late extracellular matrix (ECM) deposition.

**Methods and materials:**

We reanalyzed publicly available scRNA-seq count matrices from GSE211713 to explore microenvironmental remodeling during fibrosis. Then, we established an *in vivo* local irradiation (12 Gy) mouse model with fully irradiated wounds and boundary-spanning wounds. For tissue repair and molecular phenotyping evaluations, we used tissue staining, IHC, qPCR, and WB.

**Results:**

scRNA-seq revealed temporal decoupling between ECM-related transcription and myofibroblast function. Our mouse model showed spatial heterogeneity: although the fully irradiated wound exhibited typical inhibition of healing, the wound across the irradiation boundary showed a rapid, matrix-rich response within 14 days. TGF-β/SMAD-related signals, myofibroblast markers, and collagen deposition in the animal model were not strictly synchronous. Additionally, CellChat predicted SPP1-centered communication at the late fibrosis stage.

**Conclusions:**

Fibrosis after radiotherapy may be influenced not only by local damage but also by the boundary microenvironment. These findings support a shift toward ECM-dominant remodeling and provide a hypothesis-generating basis for targeted intervention.

## Highlights

- scRNA-seq reveals temporal decoupling between ECM-related transcription and myofibroblast function.
- A boundary-spanning wound model reveals rapid, spatially heterogeneous tissue responses.
- CellChat nominates SPP1-centered communication at the late fibrosis stage.
- Late-stage fibrosis is characterized by a shift toward ECM-dominant remodeling.

## 1 Introduction

Radiotherapy is a crucial cornerstone of multidisciplinary cancer treatment, effectively reducing tumor burden, eliminating micrometastases, and improving the local control rate of various solid tumors (1, 2). Advances in imaging techniques and precise delivery have enhanced tumor-killing efficacy while minimizing damage to normal tissues (3). For locally advanced or high-risk tumors, high-dose radiotherapy can increase tumor shrinkage, clearance, and resection rates, especially in neoadjuvant, adjuvant, and radical treatments (4–6).

However, higher doses can cause damage to normal tissues, resulting in acute erythema and edema, as well as chronic fibrosis and delayed healing (7). Clinical observations have revealed significant heterogeneity in tissue repair after radiotherapy: most patients have impaired healing, whereas others develop fibrovascular proliferation or “pseudo-grafts” composed of activated fibroblasts, endothelial cells, and excessive ECM, indicating abnormal repair processes (8–10). Rarely, radiation-induced sarcoma (RIS) may arise within chronically irradiated, fibrotic tissue (9).

Although the mechanism of radiation-induced fibrosis (RIF) has been extensively studied, the dynamic interconnection process from the activation of early damage signals to the abnormal deposition of extracellular matrix (ECM) in the later stage still lacks systematic temporal analysis (9). In this study, we identified temporal decoupling between ECM-related transcription and myofibroblast function through secondary analysis of a public single-cell RNA-sequencing (scRNA-seq) dataset (11). The original study focused on constructing a multicellular, multidose, and full-time-course map of radiation-induced lung injury. This research, however, concentrated on the dynamic evolution of fibroblast functional status, with a particular emphasis on the temporal decoupling of ECM-related transcription and myofibroblast function, as well as ECM-dominant remodeling and SPP1-centered cell communication characteristics at the late fibrosis stage. At the same time, during the establishment of a local irradiation mouse model, we unexpectedly observed an abnormal healing phenotype with high spatial heterogeneity: when the wound was completely within the irradiation area, it showed classic healing inhibition; however, when the wound crossed the boundary between the irradiation area and the non-irradiation area, the tissue exhibited a rapid focal matrix-rich response within a short period (14 days). This finding suggests that fibrosis after radiotherapy may be influenced not only by local damage but also by the microenvironment at the boundary between irradiated and non-irradiated tissues. Since the public dataset is derived from lung tissue, whereas the experimental model involves skin wounds, these two systems are treated as complementary biological contexts rather than being used for direct cross-validation.

To examine whether the “signal–matrix decoupling” pattern revealed by the public single-cell data was reflected in an independent experimental context, we used this boundary-spanning wound phenotype as an independent *in vivo* model for complementary molecular and histopathological assessment. In multiple repeated time-gradient experiments after radiotherapy, we combined IHC, Western blotting, and qPCR to evaluate the dynamic evolution of the TGF-β signaling pathway and core matrix molecules. The animal model indicates that TGF-β/SMAD-related signals, myofibroblast markers, and collagen deposition in the boundary wound are not strictly synchronized; this result is conceptually complementary to the temporal decoupling suggested by the single-cell analysis. The lung dataset and the skin wound model differ in terms of tissue type, radiation dose, injury background, and observation period. Therefore, they should be regarded as complementary rather than as a direct cross-validation.

This study combines secondary analysis of public lung single-cell data (11) with the boundary-spanning wound phenotype, highlighting the potential role of the microenvironment at the junction between irradiated and healthy tissues in determining the fate of repair, and providing a perspective for understanding temporal asynchrony between signaling and matrix remodeling in radiation-induced fibrosis.

## 2 Methods

### 2.1 Comprehensive bioinformatics analysis of single-cell transcriptomes

#### 2.1.1 Data source, quality control, dimensionality reduction, clustering, and cell type annotation of single-cell RNA sequencing data

The Seurat v5.5.0 R package was used to perform dimensionality reduction and clustering analysis on processed single-cell RNA-sequencing count matrices from GSE211713, obtained from the original murine lung radiation-injury atlas (11). To investigate changes during the early and late stages of fibrosis, five unirradiated samples were selected as the control group (Control), and two samples irradiated with 17 Gy and collected 1 month later were selected as the early fibrosis group (1M), while two samples irradiated with 17 Gy and collected 5 months later were selected as the late fibrosis group (5M). The biological sample sizes were Control = 5, 1M = 2, and 5M = 2. In the original study, SoupX was used to correct ambient RNA, and quality control was performed by excluding cells with fewer than 200 counts, a detected gene number exceeding 6,000, a mitochondrial transcript ratio exceeding 15%, or those suggesting a doublet state; since the processed matrix was used, no doublet detection was repeated. The creation parameters of the Seurat object were min.cells = 5 and min.features = 100, retaining cells with a detected gene number exceeding 100 and a mitochondrial transcript ratio below 10%. When conducting independent downstream analyses using Seurat, we adopted commonly used empirical QC thresholds and applied them uniformly to the 9 samples. Since the downloaded data had already undergone initial QC by the original authors, these thresholds were only used for the secondary screening of residual low-complexity or potentially damaged cells and were not intended to replicate, replace, or correct the QC process of the original study. A total of 58,600 cells were included: 29,906 control cells (with 7,147, 6,158, 5,561, 4,838, and 6,202 in each group, respectively), 13,404 1M cells (6,915 and 6,489, respectively), and 15,290 5M cells (7,002 and 8,288, respectively).

First, NormalizeData was used for log-normalization with a scale factor of 10,000, then FindVariableFeatures was used to identify 1,500 highly variable genes, and ScaleData was used before principal component analysis (PCA) (**Supplementary Figures 2A–C**). Harmony v2.0.2 was then applied to the PCA embeddings using orig.ident as the grouping variable to correct batch effects among samples. Based on the first 20 Harmony-corrected dimensions, the cell similarity graph was constructed using FindNeighbors, and cell clustering was performed using FindClusters (resolution = 0.5) (**Supplementary Figure 2D**). Finally, t-SNE was used for nonlinear dimensionality reduction visualization (**Supplementary Figure 2E**).

**Figure 1:**
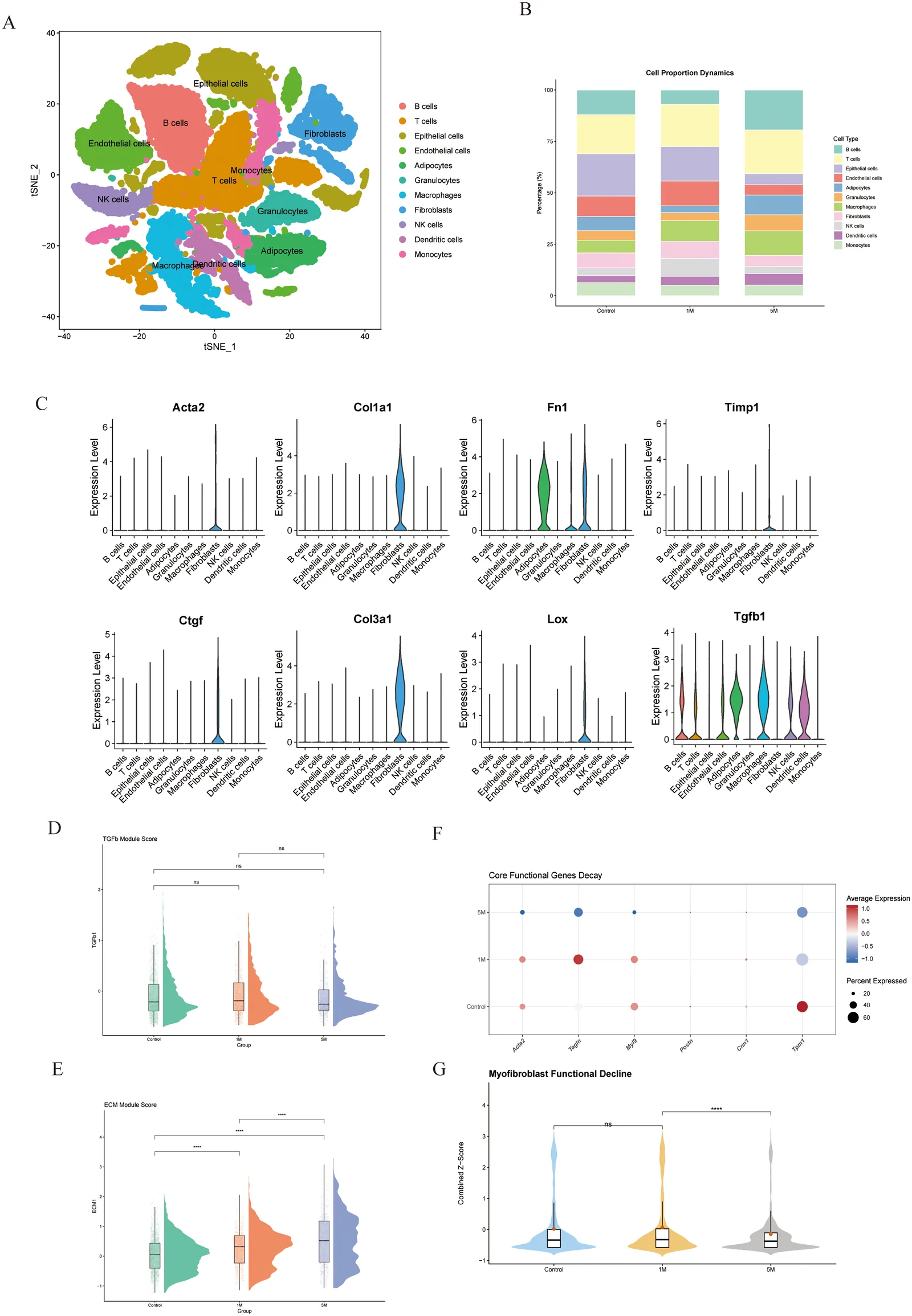
Dynamic remodeling of the whole-cell community and functional evolution of fibroblasts during different disease stages. **(A)** t-SNE clustering and cell-type annotation map of single-cell transcriptome data. **(B)** Stacked bar chart of the proportion of each cell type at different time points (Control, 1M, 5M). **(C)** Violin plot of the expression levels of fibrosis-related genes in different cell types. **(D, E)** Dynamic changes in the TGF-β signaling score **(D)** and ECM score **(E)** at different time points. Pairwise two-sided Wilcoxon rank-sum tests were adjusted within each panel using the Benjamini–Hochberg method (ns, adjusted P >= 0.05; **** adjusted P < 0.0001). **(F)** Expression level distribution of representative genes related to fibrosis and the cytoskeleton at different time points. **(G)** Changes in the Myofibroblast Function Score of fibroblasts at different time points. Pairwise two-sided Wilcoxon rank-sum tests were adjusted within the panel using the Benjamini–Hochberg method (ns, adjusted P >= 0.05; **** adjusted P < 0.0001).

Cell type annotation was carried out using a combination of automatic annotation and manual verification: first, the SingleR package was used for preliminary automatic annotation based on the MouseRNAseqData reference database (**Supplementary Figures 2F, G**); then, differentially expressed genes identified by FindAllMarkers were used, and the expression levels of classic marker genes were visualized using DotPlot (**Supplementary Figure 2H**). Finally, based on the consistency between automatic annotation results and literature reports, eleven major cell populations were manually confirmed and named (12, 13) (**Figure 1A**).

#### 2.1.2 Fibroblast subclustering and functional-state scoring

**Figure 2:**
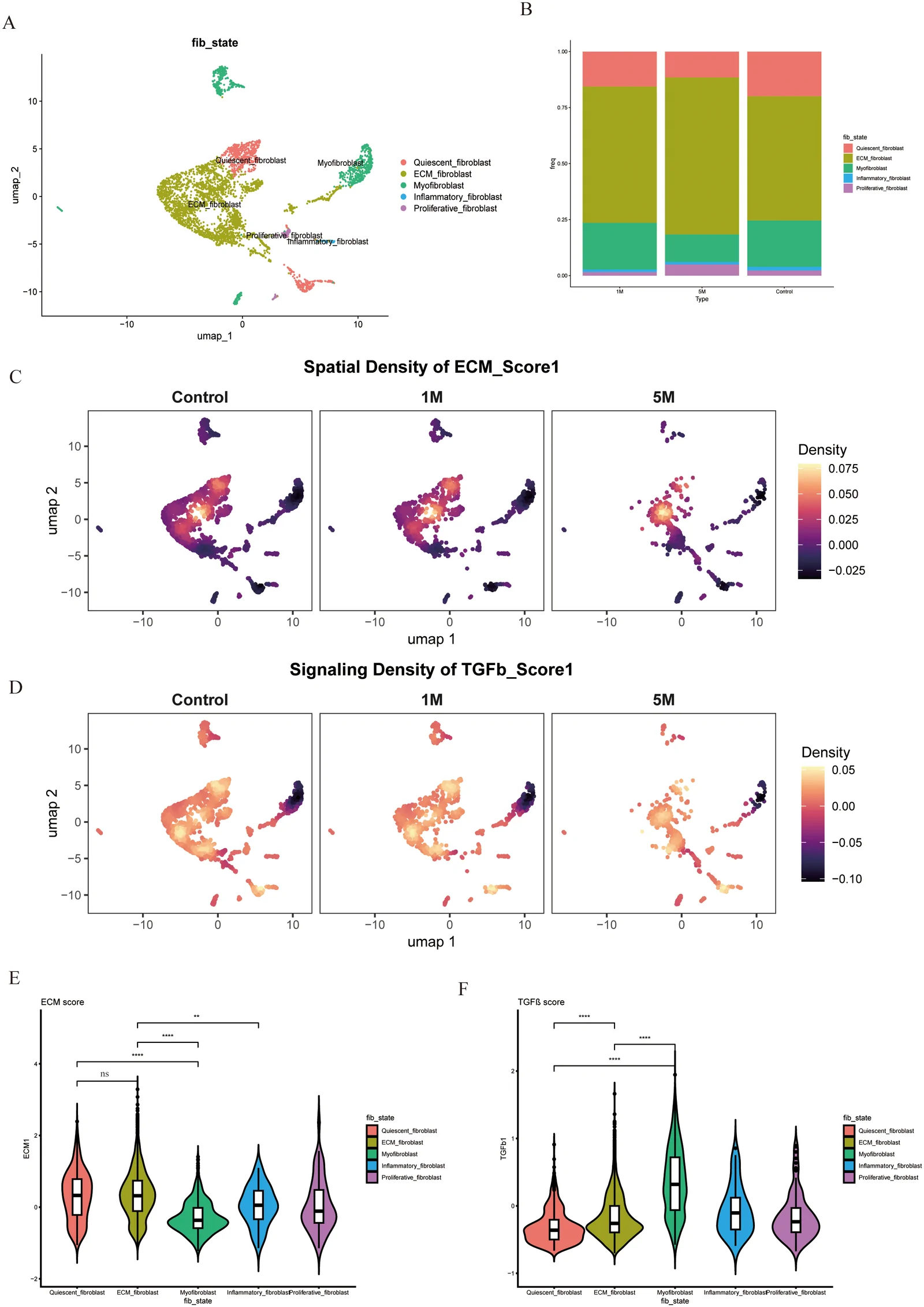
Identification of fibroblast subpopulations and analysis of their functional characteristics. **(A)** UMAP dimensionality reduction map of fibroblast re-clustering and subgroup annotation. **(B)** Composition diagram of the proportion of each fibroblast subpopulation at different time points. **(C, D)** Nebulosa UMAP embedding-density maps of the ECM and TGF-β functional modules; the panels show density in reduced-dimensional expression space rather than anatomical tissue space. **(E, F)** Quantitative comparison of ECM score **(E)** and TGF-β signaling score **(F)** among fibroblast subpopulations. Pairwise two-sided Wilcoxon rank-sum tests were adjusted within each panel using the Benjamini–Hochberg method (ns, adjusted P >= 0.05; ** adjusted P < 0.01; **** adjusted P < 0.0001).

Fibroblasts were extracted from the whole-cell map and subjected to secondary dimensionality reduction and subclustering. Clustering was performed using FindNeighbors and FindClusters (resolution = 0.4) based on the first 20 principal components (**Supplementary Figure 3A**), and UMAP was used for visualization (**Supplementary Figure 3B**). Combined with DotPlot results of classical marker gene expression (**Supplementary Figure 3C**), five fibroblast subpopulations with different biological functions were finally annotated: quiescent state (Dcn/Lum^+^), ECM-producing state (Col1a1/Timp1^+^), myofibroblast (Acta2/Tagln^+^), inflammatory state (Cxcl12/Ccl2^+^), and proliferative state (Mki67^+^) (14, 15) (**Figure 2A**).

Further, the ECM gene set (Col1a1, Col3a1, Fn1, Lox, and Timp1) (16) was selected with reference to published radiation-associated ECM-remodeling studies; the corresponding ECM score was calculated using AddModuleScore. This set can measure fibrillar collagen abundance, matrix assembly, collagen cross-linking, and matrix turnover. The TGF-β signaling score (17) (Tgfb1, Smad2, Smad3, Ctgf, Serpine1, and Acta2) was calculated in parallel (**Figures 1D, E**, **2E, F**). The TGF-β gene set represents the ligand-SMAD-downstream response axis described in the published pathway studies. The ECM and TGF-β scores were custom, literature-guided modules rather than curated pathway-database signatures. To demonstrate the complementary biological characteristics related to myofibroblasts, six genes were selected for visualization at the gene level: Acta2, Tagln, Myl9, Cnn1, Tpm1 (tropomyosin 1), and Postn (periostin). Among them, Acta2, Tagln, Myl9, and Cnn1 encode functions related to actin-myosin. When conducting a comprehensive functional assessment of myofibroblasts, only Acta2, Tagln, Myl9, and Cnn1 were included to maintain consistency in function. Postn was excluded because it mainly reflects secreted extracellular matrix products, while the gene expression level of Tpm1 cannot distinguish between different splicing isoforms, which have different roles in actin filament and myosin functions (18–22). Acta2, Tagln, Myl9, and Cnn1 were Z-score normalized by row and averaged at the single-cell level to define the Myofibroblast Function Score (**Figure 1G**). This score was used as a descriptive composite index and is not a validated clinical biomarker. Row-wise normalization reduced bias caused by absolute differences in gene expression. Module-score distributions in UMAP expression space were evaluated using Nebulosa (**Figures 2C, D**).

#### 2.1.3 Single-cell trajectory inference and temporal sequence analysis

**Figure 3:**
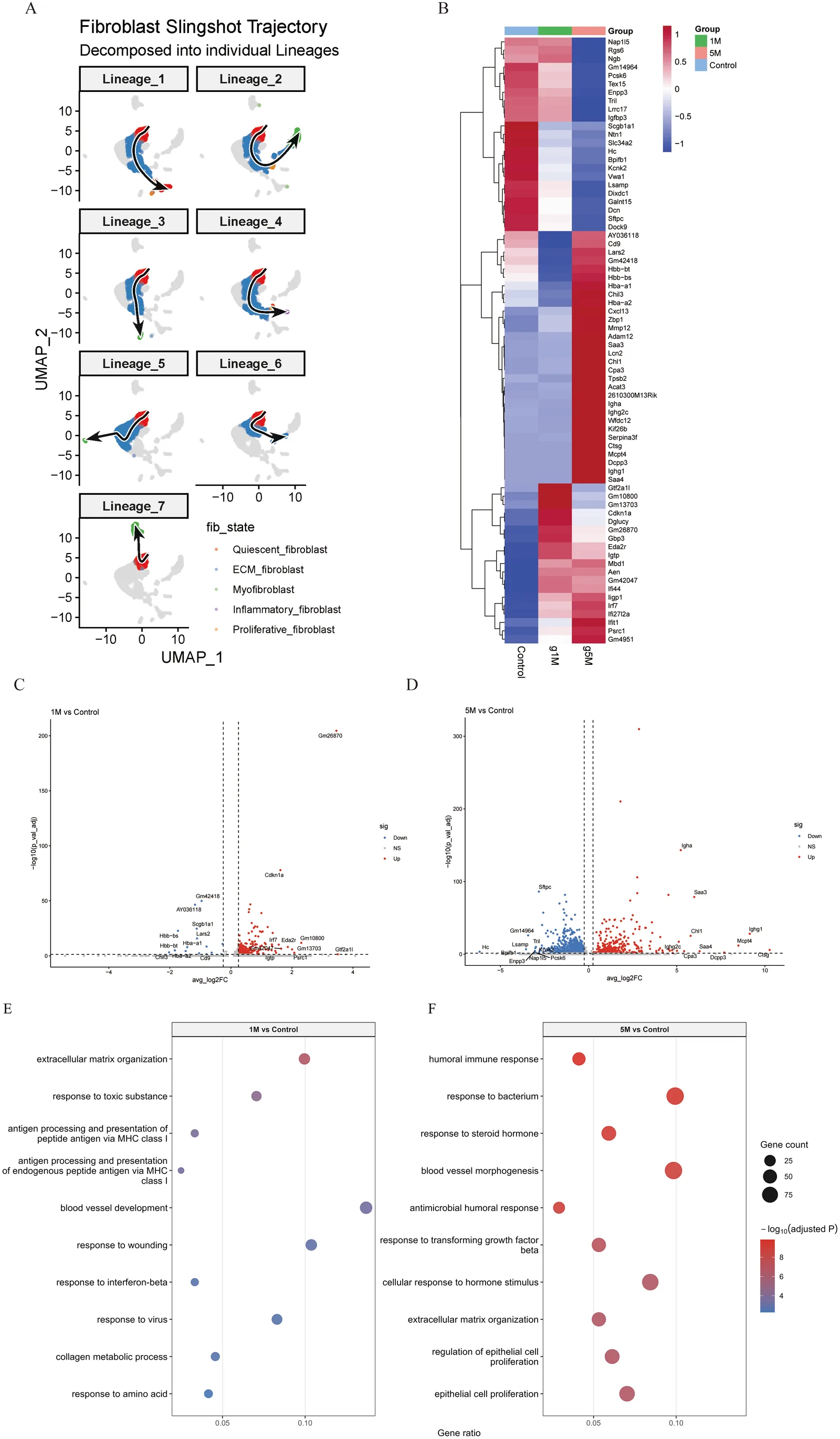
Inferred fibroblast differentiation trajectories, time-point-specific gene expression, and GO Biological Process enrichment. **(A)** Projection of seven computationally inferred fibroblast-state branches in UMAP space using Slingshot; these paths represent model-based state relationships rather than direct lineage tracing. **(B)** Heatmap of specifically highly expressed genes in fibroblasts at different time points (Control, 1M, 5M). **(C)** Volcano plot of differentially expressed genes in the 1M stage compared to the Control group, with the top 10 significantly upregulated and downregulated genes annotated. **(D)** Volcano plot of differentially expressed genes in the 5M stage compared to the Control group, with the top 10 significantly upregulated and downregulated genes annotated. **(E)** GO Biological Process enrichment of all significant fibroblast DEGs at 1M compared with Control. **(F)** GO Biological Process enrichment of all significant fibroblast DEGs at 5M compared with Control. For panels E-F, DEGs were defined using adjusted P < 0.05 and |avg_log2FC| > 0.25; upregulated and downregulated genes were analyzed together; all genes tested in the corresponding comparison were used as the background; and GO terms with Benjamini-Hochberg-adjusted P < 0.05 were considered significant. The top 10 nonredundant terms are shown; dot size represents gene count, and color represents -log10(adjusted P).

To explore the developmental and differentiation trajectories of fibroblasts, this study used the Slingshot v2.18.0 R package for single-cell developmental trajectory inference. First, the Seurat object was converted to a SingleCellExperiment object, and UMAP dimensionality reduction coordinates were extracted. Based on previous clustering results, Cluster 1 was designated as the starting cluster of quiescent fibroblasts (Quiescent fibroblasts), and multi-branch trajectory inference was performed. To avoid signal confusion caused by multi-branch mixing, pseudotime of cells was calculated for each lineage separately. Dynamic changes in gene expression trends were fitted using a generalized additive model (GAM) and displayed by branches. The proportion of cell state succession was obtained by dividing each branch pseudotime into 20 bins and calculating the proportion of cell states. The direction and flow of the trajectory were visualized using the ggarrow package, which intuitively reflected the differentiation path in arrow form, and the patchwork package was used for multi-figure joint layout (**Figure 3A**). These trajectories represent computationally inferred state transitions rather than direct lineage tracing.

Guided by published descriptions of fibrotic fibroblast states, this study examined representative genes—Col1a1 and Fn1 for ECM production and assembly, Tagln and Acta2 for contractile activation, and Ccl2 for inflammatory signaling—to characterize these functions along the seven lineages inferred by Slingshot (23). Dynamic expression changes of each gene over the inferred time sequence were locally smoothed and fitted using a generalized additive model (GAM, formula: y ~ s(x)), and were independently presented on a branch-by-branch basis (**Supplementary Figure 3D**).

#### 2.1.4 Differential expression analysis of fibrosis-associated genes and GO biological process enrichment

After setting the group identities in the fibroblast dataset, the FindMarkers function was used to conduct differential expression analysis between 1M vs Control and 5M vs Control, with screening criteria of adjusted P value < 0.05 and |log2FC| > 0.25. Fibrosis-associated differentially expressed genes were obtained and the results were exported. Based on the results of the differential analysis, a volcano plot was constructed, dividing genes into up-regulated, down-regulated, and not significantly different categories, and marking the top 10 up-regulated and down-regulated genes with the most significant log2FC changes, to display the expression changes of key genes in disease progression (**Figures 3C, D**). Subsequently, using clusterProfiler v4.18.4 and org.Mm.eg.db v3.22.0 v3.22.0, a GO biological process over-representation analysis was conducted for all significantly differentially expressed genes (DEGs) in each comparison. Upregulated and downregulated genes were analyzed together, and all genes tested in the corresponding comparison were used as the background. GO terms with Benjamini–Hochberg-adjusted P values < 0.05 were considered significant; redundant terms were removed based on semantic similarity (threshold = 0.7), and the top 10 non-redundant GO terms were displayed (**Figures 3E, F**).

#### 2.1.5 Heatmap analysis of key differentially expressed genes in fibrosis

Further, significantly up-regulated and down-regulated genes (|log2FC| > 0.5, adjusted P value < 0.05) were selected from the results of the two-group differential analysis, and the top 20 genes from each group were selected and merged to construct a heatmap gene set. The AverageExpression function was used to calculate the average expression matrix of each group, and gene expression was standardized by Z-score row normalization to eliminate differences in gene expression magnitude. Finally, a pheatmap clustering heatmap was drawn according to the time sequence of Control, 1M, and 5M, and annotations of groups were added to display the dynamic expression patterns of fibrosis-associated genes during disease progression (**Figure 3B**).

#### 2.1.6 Cell communication network analysis and key ligand SPP1 signal screening

**Figure 4:**
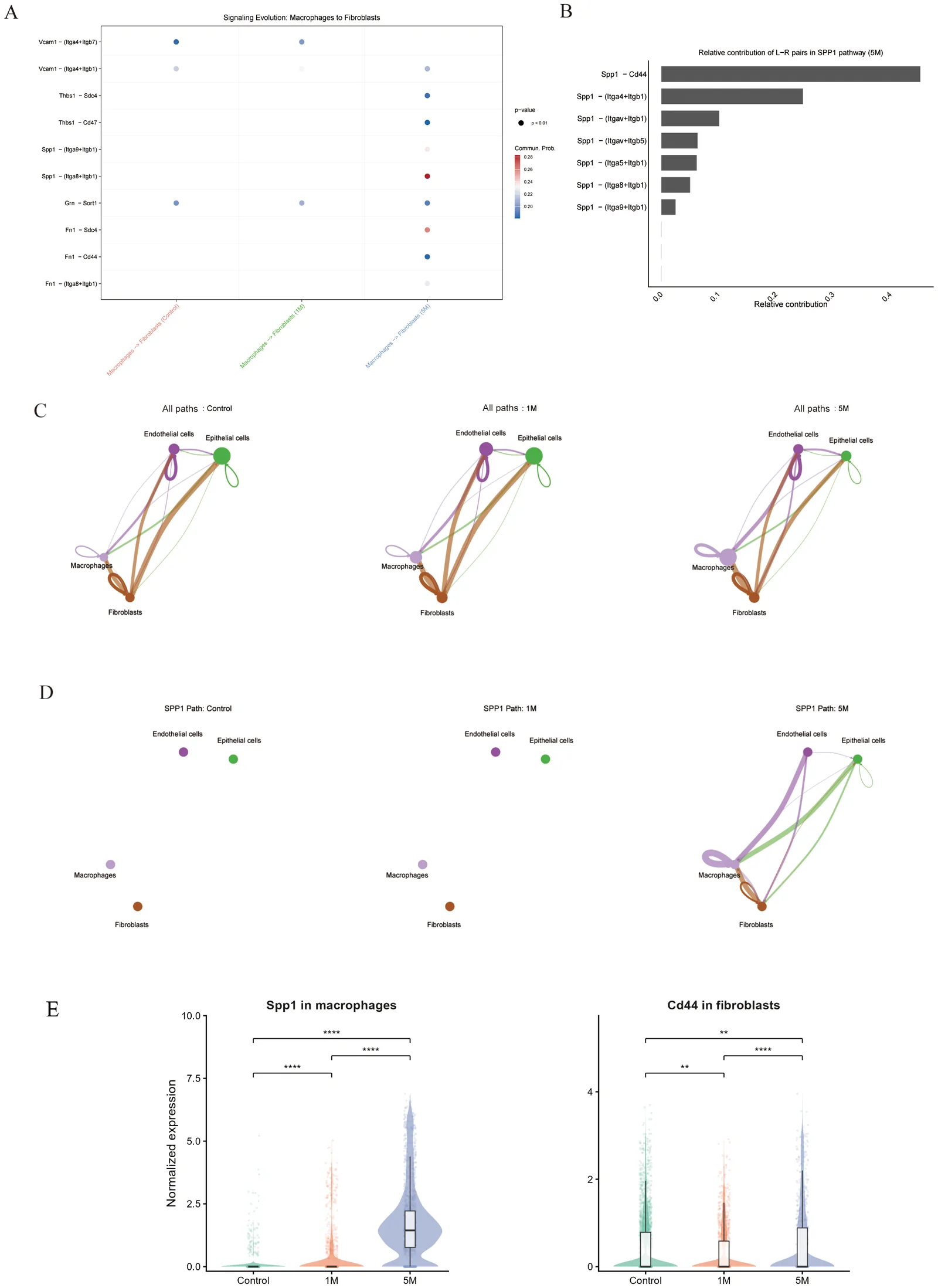
Identification of cell communication networks and SPP1 signaling pathway at different disease stages. **(A)** Bubble plot showing macrophage-to-fibroblast ligand–receptor communication across Control, 1M, and 5M; detected 5M SPP1 interactions include Spp1–(Itga8+Itgb1) and Spp1–(Itga9+Itgb1). **(B)** Relative contribution of ligand–receptor pairs in the complete 5M SPP1 signaling pathway across all analyzed cell populations; this panel is not restricted to macrophage-to-fibroblast communication. **(C)** Network plot depicting the overall number and intensity of intercellular communication in the microenvironment at different time points (Control, 1M, 5M). **(D)** Comparison of dynamic changes in cell communication networks of the SPP1 signaling pathway at different time points (Control, 1M, 5M). **(E)** Violin plots showing normalized Spp1 expression in macrophages and Cd44 expression in fibroblasts across Control, 1M, and 5M. Small points represent individual cells. Pairwise annotations are based on two-sided Wilcoxon rank-sum tests with Benjamini–Hochberg correction within each expression panel. ** adjusted P < 0.01; **** adjusted P < 0.0001.

Cell communication analysis was performed separately for Control, 1M, and 5M. Core populations such as macrophages, fibroblasts, endothelial cells, and epithelial cells were included to construct condition-specific objects using CellChat v1.6.1 and CellChatDB.mouse. Communication probabilities were calculated from normalized RNA expression using computeCommunProb (raw.use = TRUE, population.size = FALSE, nboot = 100, seed.use = 1). All retained cells were used without downsampling, and the three objects were merged only for cross-stage visualization. Because cell numbers differed among groups, the results were interpreted as relative estimates. Accordingly, the CellChat results were considered hypothesis-generating. Significant interactions were extracted using thresh = 0.05 (**Figure 4C**) (24).

To systematically screen signal inputs from macrophages to fibroblasts at 5M, subsetCommunication was used with sources.use = “Macrophages” and targets.use = “Fibroblasts,” and interactions were ranked by communication probability and permutation P value (**Figure 4A**). **Figure 4B** shows ligand–receptor contributions across the complete 5M SPP1 pathway without source or target restriction. Normalized Spp1 expression in macrophages and Cd44 expression in fibroblasts was additionally examined across conditions (**Figure 4E**).

### 2.2 Establishment of animal models and phenotypic analysis

#### 2.2.1 Animal husbandry and ethical considerations

BALB/c mice (6–8 weeks, 22–25 g) of both sexes (purchased from Biocytogen, Wuhan Biocytogen Biotechnology Co., Ltd.) were housed under SPF conditions. Mice were used as mixed-sex cohorts and randomly assigned to four groups (n = 6 per time point); sex was not used as a stratification factor, and sex-specific effects were not analyzed.

Wound creation was performed under 2% isoflurane inhalation anesthesia to ensure full unconsciousness during the procedure. Animals were monitored daily for general health, body weight, wound appearance, activity, and behavior. Humane endpoints were predefined to prevent unnecessary suffering. Mice were euthanized immediately if they exhibited severe ulceration or necrosis, inability to eat or drink, persistent lethargy, or other signs of severe distress prior to the scheduled endpoint.

All mice were euthanized 14 days after wound creation by cervical dislocation performed under deep anesthesia with isoflurane, and tissues were collected for histological and immunohistochemical analyses. No animals reached humane endpoints before the planned termination of the experiment.

#### 2.2.2 Experimental grouping and design

To study the dynamic process of wound healing after radiotherapy, mice were randomly divided into four groups (n = 6 per group at each time point):

Fully irradiated wound model (W group): The back of the mice was pre-exposed to 12 Gy of local high-dose radiation (irradiation diameter 1 cm) (25, 26), and then a 6 mm full-thickness circular wound was created with its center aligned to the center of the irradiated field to simulate the typical pattern of post-radiotherapy healing disorder (27).

Boundary-spanning wound model (H group): The center of a 6 mm full-thickness circular wound was positioned on a marked point along the circumference of the 1 cm-diameter irradiation field. Because this placement positioned the wound approximately half within the irradiated field and half in adjacent non-irradiated tissue, it was designated the H group; this was not an exact half-and-half division and was defined by geometric placement rather than measured edge dosimetry.

Within-animal validation model (V group): In the same mouse and under the same overall irradiation background, both H-type and W-type wounds were established to provide within-animal validation of the contrast between the two wound configurations. V-group images were used to validate the within-animal contrast qualitatively; it was not treated as an independent validation cohort or included in pooled H/W inferential analyses.

Non-irradiated control group (NC group): A full-thickness circular wound was prepared on the normal unirradiated back skin.

#### 2.2.3 Local irradiation and wound preparation

Local irradiation was performed using the SMART+ Irradiation System (Precision X-Ray, USA), with a dose rate of approximately 3 Gy/min and a cumulative dose of 12 Gy (25). The rest of the mouse body was shielded with a 3 mm thick lead plate to ensure that the irradiation area was limited to the specified region (28). Before irradiation, the circumference of the 1 cm-diameter field was marked on the dorsal skin. Wound-scale dose mapping across the field edge and quantitative shielding-scatter measurements were not performed.

Wounds were established at 1, 5, 9, and 13 days after irradiation (designated as R1, R5, R9, and R13 time points). The back of the mice was prepared with a 6 mm sterile biopsy punch under 2% isoflurane inhalation anesthesia. During the first 2 days post-surgery, the wounds were covered with 3M™ Tegaderm™ Transparent Film to prevent infection (29). For H wounds, the punch center was aligned with the premarked field circumference; for W wounds, the entire wound outline was positioned within the marked field.

#### 2.2.4 Wound observation and histological analysis

Wound photos were taken every other day after the operation, and the observation lasted for 14 days (30, 31). The wound area ratio was calculated using standardized image analysis methods (current wound area/wound area on day 0 × 100%) to evaluate the wound closure speed. Histological regions of interest were defined consistently across groups. No formal blinding procedures were recorded for wound area measurement or histological quantitative analysis, so these analyses were non-blinded.

On day 14 after the operation, wound tissues were collected for H&E staining, Masson staining, and immunohistochemical analysis. The IHC antibody identifiers and working dilutions are specified in Section 3.2.

For systematic analysis, the tissues of each group were mixed according to the experimental design: NC group tissues were mixed and recorded as the NC group; R1H, R5H, R9H, and R13H tissues were mixed and recorded as the H group; R1W, R5W, R9W, and R13W tissues were mixed and recorded as the W group. The mixed tissues were then used for qPCR and Western blot detection to comprehensively evaluate the changes in cell proliferation, angiogenesis, inflammatory response, and fibrosis-related indicators in the wound. These pooled molecular assays were descriptive only and were not used for animal-level inference.

### 2.3 Histological and immunohistochemical staining

Wound tissues were harvested at predetermined time points, fixed in 4% paraformaldehyde (PFA) for over 24 hours, and then embedded in paraffin and cut into 4 μm consecutive sections for histological and molecular evaluation. The paraffin-embedded tissue sections (4 μm) were dewaxed in warm water and adhered to glass slides.

#### 2.3.1 Hematoxylin–eosin and Masson staining

H&E staining and Masson staining were used to evaluate the degree of skin tissue injury and fibrosis. H&E staining was performed according to conventional methods to observe tissue morphology, structure, and inflammatory cell infiltration. Masson staining was used to detect collagen deposition and the degree of fibrosis, and the procedure was strictly performed in accordance with the kit instructions.

#### 2.3.2 Immunohistochemistry staining

IHC staining detected the expression and localization of target proteins through antigen–antibody binding. First, the sections were dewaxed with xylene I/II for 10 minutes each, then gradually hydrated with 100%, 95%, 85%, and 70% ethanol. The sections were incubated with 20 μg/mL proteinase K at 20–37 °C for 15–30 minutes for antigen retrieval. After PBS washing, the sections were immersed in 3% H2O2 for 10 minutes to inactivate endogenous peroxidase, and then incubated with blocking solution at room temperature for 10 minutes to block non-specific binding.

The primary antibodies were anti-Ki67 (Abcam, Cambridge, UK; ab16667; 1:200), anti-CD31 (Abcam, Cambridge, UK; ab182981; 1:2,000), anti-CD68 (Abcam, Cambridge, UK; ab125212; 1:2,000), anti-α-SMA (Abcam, Cambridge, UK; ab124964; 1:1,000), anti-TGF-β1 (Abcam, Cambridge, UK; ab215715; 1:500), anti-Smad2/3 (Abcam, Cambridge, UK; ab305325; 1:250), and anti-COL1A1 (Abcam, Cambridge, UK; ab88147; 1:200). They were diluted as indicated and incubated at room temperature for 1–2 hours, then washed with PBS. The corresponding secondary antibodies were added and incubated at room temperature for 30 minutes, followed by washing. Staining was performed using DAB reagent under a microscope, and the staining process was monitored. The reaction was terminated with PBS. After staining, the results were evaluated based on the expression intensity of the target protein and the background signal. The slides were digitized using a PANNORAMIC MIDI II whole-slide scanner (3DHISTECH Ltd., Budapest, Hungary) equipped with a 20× objective lens. The whole-slide images were reviewed using CaseViewer version 2.4 (3DHISTECH Ltd., Budapest, Hungary), and quantitative analysis was performed using ImageJ software (National Institutes of Health, Bethesda, MD, USA). In the analyzed regions of interest, CD31 was expressed as the number of blood vessels per field of view; Ki67 was expressed as the percentage of positive nuclei; CD68, TGF-β1, Smad2/3, and α-SMA were expressed as the percentage of DAB-positive area; COL1A1 was expressed as integrated optical density (IOD); and Masson staining was expressed as the percentage of collagen-positive area. Multiple regions within the same tissue section were regarded as technical subsamples rather than independent biological replicates.

### 2.4 Molecular biology assays

qPCR: RNA was extracted from wound tissues, and the expression of genes related to inflammation, tissue repair, and fibrosis (e.g., CCL2, SERPINE1, PDGFB, AREG) was analyzed. RNA was extracted using TRIzol reagent (Servicebio, Wuhan, China), and the concentration was measured with a NanoDrop spectrophotometer. cDNA was synthesized in a 20 μL system using a reverse transcription kit (Biosharp, Hefei, China) and stored at −80 °C.

qRT-PCR was performed on a fluorescence quantitative PCR instrument (Monad Biotech, Wuhan, China). GAPDH was used as the internal reference and relative expression levels were analyzed using the 2^–^ΔΔCt method. Primer sequences are listed in the **Supplementary Materials** (**Supplementary Table S1**).

Western blotting: Protein levels of TGF-β, α-SMA, and CD68 were measured. Skin tissue was homogenized on ice in RIPA buffer containing 1 mM PMSF and centrifuged at 12,000 × g for 10 minutes at 4 °C. After protein concentration was determined using the BCA method, equal protein amounts were separated by SDS–PAGE and transferred to PVDF membranes. The primary antibodies were anti-TGF-β1 (Abcam, Cambridge, UK; ab215715; 1:1,000), anti-α-SMA (Abcam, Cambridge, UK; ab124964; 1:10,000), anti-CD68 (Abcam, Cambridge, UK; ab125212; 1:2,000), and anti-GAPDH (Abcam, Cambridge, UK; ab8245; 1:5,000). Membranes were blocked with 5% skimmed milk for 2 hours at room temperature, incubated with primary antibody overnight at 4 °C and HRP-conjugated secondary antibody (1:8,000) for 2 hours, and developed using ECL. Band intensities were quantified using ImageJ (National Institutes of Health, Bethesda, MD, USA), normalized to GAPDH, and expressed as target protein/GAPDH ratios. Full uncropped blots are provided in **Supplementary Figure 1**. Because NC, H, and W tissues were pooled at the group level, each lane represents one pooled sample and densitometric values were descriptive rather than inferential.

### 2.5 Statistical analysis

Statistical analyses were conducted using R v4.5.2. Cell-level module scores in **Figures 1D, E, G**, and **2E, F** were compared using unpaired two-sided Wilcoxon rank-sum tests. Only the P values for the pairwise comparisons shown in the figures were adjusted using the Benjamini–Hochberg (BH) method within each panel; no additional correction was applied across different panels. **Figure 1F** is a descriptive DotPlot, and no hypothesis test was conducted for this panel. The potential for pseudoreplication in cell-level analyses is addressed in the Limitations section.

All single-cell differential expression analyses based on the Seurat FindMarkers function employed the default two-sided Wilcoxon rank-sum test, and the resulting P values were adjusted using Seurat’s default Bonferroni method. For the GO biological process over-representation analysis in **Figures 3E, F**, the hypergeometric test implemented in clusterProfiler was used to evaluate enrichment; P values were adjusted using the BH method, and adjusted P values < 0.05 were considered significant. The significance of CellChat interactions was evaluated using permutation P values, with P < 0.05 used as the screening threshold. In **Figure 4E**, single-cell normalized Spp1 expression in macrophages and Cd44 expression in fibroblasts was compared between groups using unpaired two-sided Wilcoxon rank-sum tests. The P values from the three pairwise comparisons within each expression panel were adjusted using the BH method.

**Figure 5:**
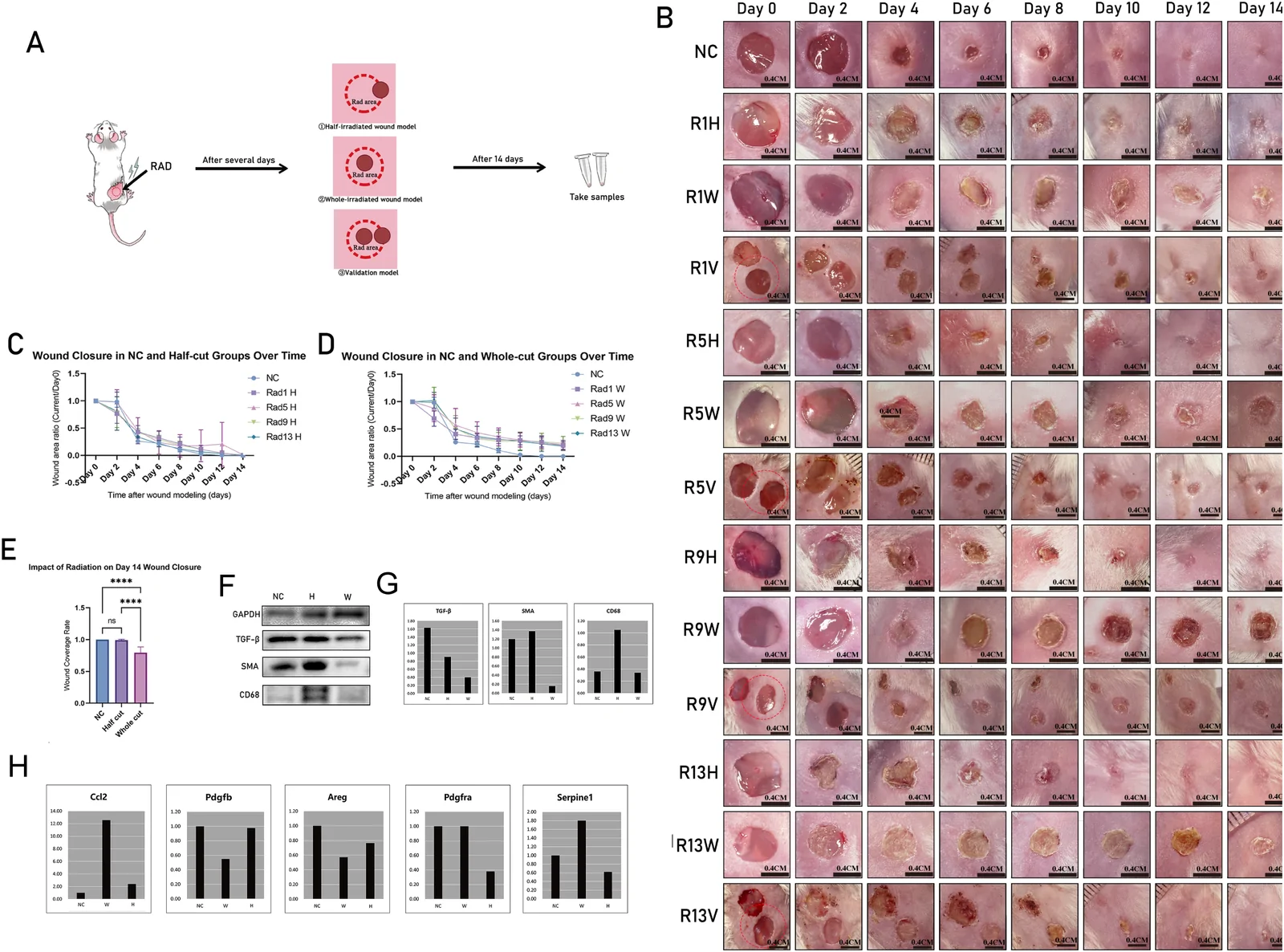
Impact of localized irradiation on wound healing in mice. **(A)** Experimental workflow: Mice received localized irradiation (12 Gy, 1 cm diameter), followed by 6-mm full-thickness wounds at 1, 5, 9, or 13 days after irradiation (R1, R5, R9, R13). In the boundary-spanning H configuration, the wound center was placed on the field circumference so that the wound approximately crossed irradiated and non-irradiated tissue; this was not an exact half-and-half division. In the W configuration, the wound was fully contained within the irradiated field. The within-animal validation model (V group) combined boundary-spanning H and fully irradiated W wounds in the same mouse to validate the contrast between the two configurations. Wounds were collected 14 days after surgery for histology. **(B)** Representative wound images recorded every 2 days (days 0–14). Wounds were covered with 3M Tegaderm for the first 2 days. **(C, D)** Wound closure kinetics over time: wound area ratios (current/initial) in NC versus boundary-spanning H wounds **(C)** and NC versus fully irradiated W wounds **(D)**. Data are mean ± SEM; these longitudinal panels are descriptive. **(E)** Wound closure at day 14 (1 − wound area/day 0), summarized for NC, pooled H, and W groups (n = 6 animals per irradiation-to-wounding interval). ns, P >= 0.05; **** P < 0.0001. **(F)** Western blot analysis of TGF-β, α-SMA, and CD68 under NC, boundary-spanning H, and fully irradiated W conditions; each lane represents one pooled group-level tissue sample. **(G)** Quantification of Western blot bands: TGF-β, α-SMA, and CD68 intensities were normalized to GAPDH; each group was represented by one pooled tissue sample, so the densitometric values are descriptive. **(H)** Descriptive qPCR analysis of repair-related genes in pooled NC, fully irradiated W, and boundary-spanning H tissue samples.

**Figure 6:**
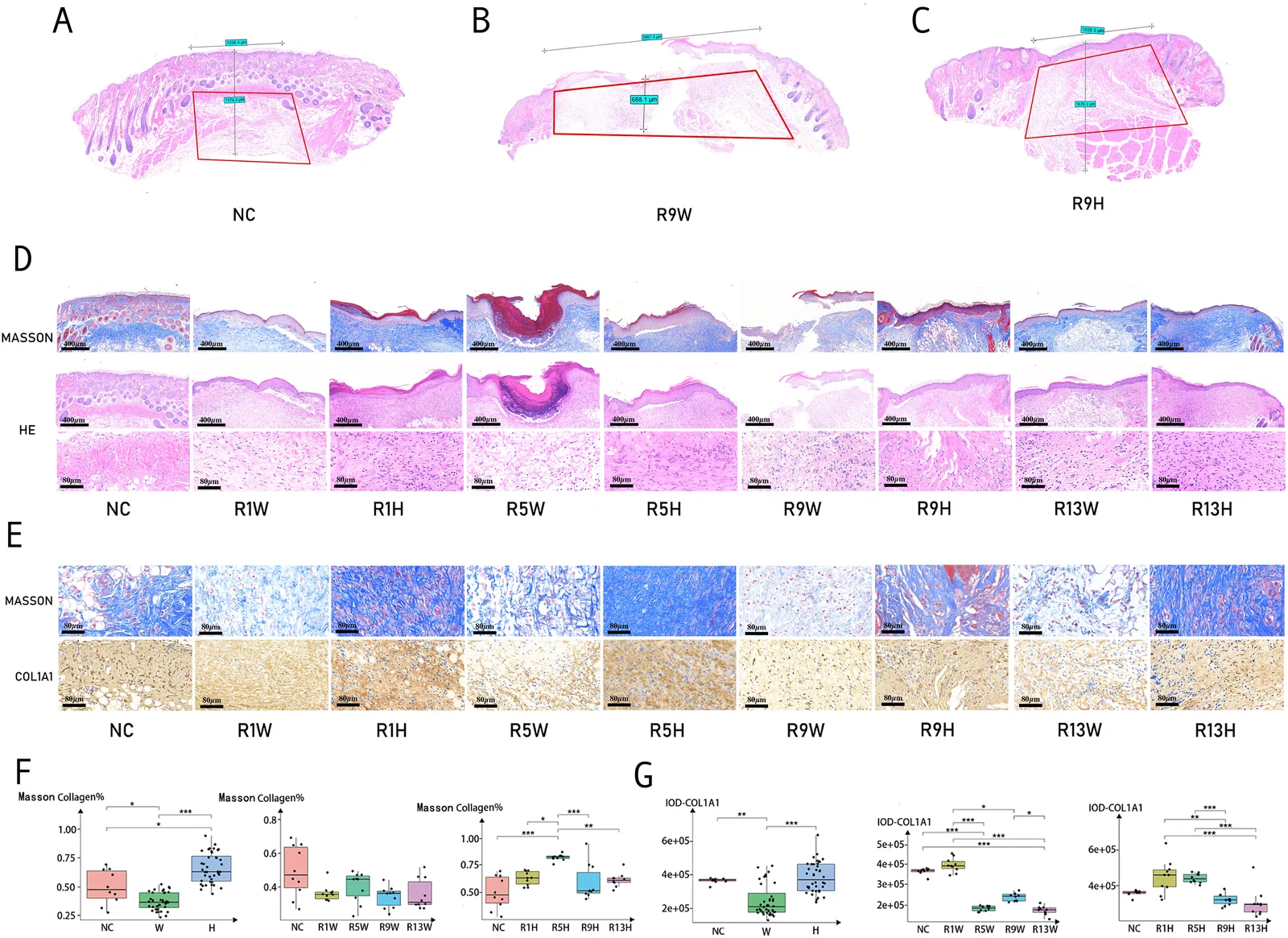
Histological analysis of wound healing following localized irradiation in mice: collagen deposition and COL1A1 expression. **(A–C)** HE panoramic images of NC, R9W, and R9H wounds; red boxes indicate dermal regions analyzed. **(D)** Representative images at different magnifications: 10× HE and Masson (epidermis) and 40× HE (dermis). **(E)** Masson trichrome staining and COL1A1 immunohistochemistry in NC, boundary-spanning H, and fully irradiated W wounds. **(F, G)** Quantification of collagen deposition and COL1A1 expression. Multiple regions of interest were quantified per tissue section; n = 6 animals per irradiated configuration at each irradiation-to-wounding interval. R1, R5, R9, and R13 indicate wounds created 1, 5, 9, and 13 days after irradiation, respectively; all tissues were collected 14 days after wound creation. Statistical methods are described in the Statistical Analysis section.

**Figure 7:**
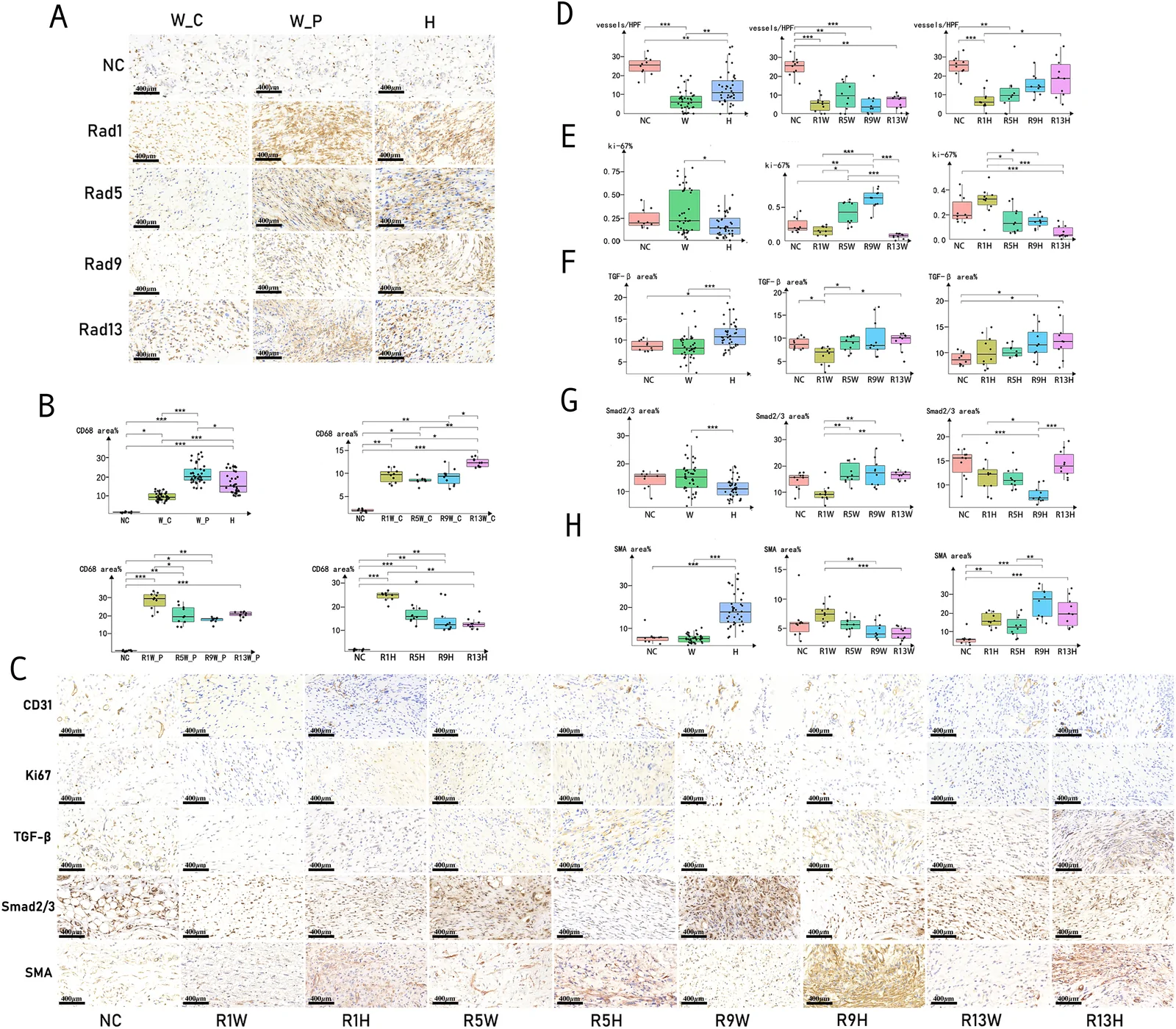
Spatial and temporal distribution of macrophages and immunohistochemical analysis of wound healing. **(A, B)** CD68 immunostaining and quantification showing the regional and temporal distribution of macrophages. Regions: NC = non-irradiated control, W_C = central area of a fully irradiated wound, W_P = peripheral area of a fully irradiated wound, H = boundary-spanning wound. **(C)** Representative immunohistochemistry images for CD31, Ki67, TGF-β, SMAD2/3, and α-SMA. **(D–H)** Quantitative analysis of CD31, Ki67, TGF-β, SMAD2/3, and α-SMA in NC, boundary-spanning H, and fully irradiated W wounds over time. Multiple regions of interest were quantified per tissue section; n = 6 animals per irradiated configuration at each irradiation-to-wounding interval. R1, R5, R9, and R13 indicate wounds created 1, 5, 9, and 13 days after irradiation, respectively; all tissues were collected 14 days after wound creation. * adjusted P < 0.05; ** adjusted P < 0.01; *** adjusted P < 0.001.

Unless otherwise stated, animal experimental data are presented as the mean ± standard error of the mean (SEM). The longitudinal wound-area curves in **Figures 5C, D** are descriptive, and no hypothesis tests were conducted for these panels. Day 14 was the prespecified primary endpoint for inferential comparison; the wound-closure rate in **Figure 5E** was analyzed using one-way analysis of variance (ANOVA), followed by Tukey’s multiple-comparisons test. Histological and immunohistochemical data in **Figures 6F, G** and **7B, D–H** were analyzed using the Kruskal–Wallis test, followed by Dunn’s pairwise comparisons with BH adjustment of the P values. Multiple image fields or regions of interest within each tissue section were technical subsamples from the same animal and did not constitute additional independent biological replicates. Western blot band intensities in **Figures 5F, G** were quantified using ImageJ and normalized to GAPDH; together with the qPCR results in **Figure 5H**, these pooled group-level readouts were interpreted descriptively without inferential statistical testing.

## 3 Results

### 3.1 During the progression of the disease, the dynamic remodeling of the whole-cell profile and the functional evolution of fibroblasts

The clustering and annotation results of the single-cell data are shown in **Figure 1A**. To clarify the dynamic remodeling process of the cellular microenvironment at different time points (Control, 1M, 5M), we first analyzed the cell composition at the whole-cell level. The stacked bar chart results indicated that fibroblasts, endothelial cells, and epithelial cells maintained relatively stable proportions in the Control and 1M stages but showed lower proportions at 5M. In contrast, the proportions of adipocytes and immune cell populations (such as macrophages, DCs, T cells, etc.) showed a continuous upward trend with disease progression and reached a peak at the 5M stage; moreover, the proportion of B cells decreased first and then increased, while NK cells showed an increase first and then a decrease (**Figure 1B**). Further, by analyzing the expression of fibrosis-related genes in various cells, the violin plot results confirmed that these genes were specifically expressed in fibroblasts (**Figure 1C**).

By performing module score analysis on fibroblast subpopulations, we observed stage-associated differences in different functional modules. After Benjamini–Hochberg correction, no pairwise differences in the TGF-β signaling score were significant (**Figure 1D**). In contrast, the ECM score was higher at 1M and 5M than Control and higher at 5M than 1M (all adjusted P < 0.0001; **Figure 1E**). The four genes included in the score (Acta2, Tagln, Myl9, and Cnn1) showed higher expression at 1M than at 5M, whereas Postn and Tpm1 displayed non-concordant gene-specific patterns and were therefore retained only in the descriptive DotPlot (**Figure 1F**). Separately, after correction, the four-gene Myofibroblast Function Score did not differ between 1M and Control but was lower at 5M than at 1M (adjusted P < 0.0001; **Figure 1G**).

### 3.2 Identification of fibroblast subpopulation heterogeneity and analysis of pathogenic potential

Considering that different fibroblast subpopulations may have distinct pathogenic potentials, we extracted fibroblasts from the whole-cell map and performed subgroup classification and annotation (**Figure 2A**). The cell proportion analysis showed that at 5M the proportion of ECM fibroblasts increased, whereas the proportion of myofibroblasts decreased (**Figure 2B**). ECM scores did not differ between ECM and quiescent fibroblasts but were higher in both groups than in myofibroblasts (both adjusted P < 0.0001), and ECM fibroblasts also had higher scores than inflammatory fibroblasts (adjusted P = 0.0010). TGF-β signaling scores differed in all displayed comparisons among quiescent, ECM, and myofibroblast states (all adjusted P < 0.0001), with the highest scores in myofibroblasts (**Figures 2E, F**). Nebulosa maps showed the distributions of the ECM and TGF-β signaling scores across the fibroblast UMAP (**Figures 2C, D**).

### 3.3 Inference of fibroblast differentiation trajectory and disease stage-specific transcriptomic features

To investigate the state heterogeneity of fibroblasts during disease progression or in steady-state conditions, this study used the Slingshot algorithm to infer state trajectories. We set Cluster 1 as the starting point of the inferred trajectories. The results showed that the cell population presented seven distinct differentiation branches (Lineages). In the UMAP dimensionality reduction space, each branch represented a model-inferred state relationship from the quiescent starting cluster toward different functional regions (**Figure 3A**): Lineages 1–6 started from the quiescent state and first pointed to the ECM-enriched subpopulation (as a potential intermediate state); then Lineages 2, 3, and 5 extended toward the myofibroblast region; Lineage 4 extended toward the inflammatory fibroblast region; Lineage 1 extended toward another quiescent-state region; moreover, Lineage 7 extended from the quiescent state toward the myofibroblast region (**Figure 3A**). Characteristic-gene expression trends were generally concordant with the inferred state trajectories and are presented as supportive, exploratory evidence (**Supplementary Figure 3D**).

Furthermore, we conducted differential gene expression analysis of fibroblasts at different stages. Compared with the Control group, 263 significant DEGs were identified at 1M and 1,065 at 5M (**Figures 3C, D**). Formal GO Biological Process over-representation analysis was then performed separately for the two comparisons (**Figures 3E, F**). At 1M, significant terms included extracellular matrix organization, response to wounding, blood vessel development, collagen metabolic process, response to interferon-beta, and response to virus (**Figure 3E**). At 5M, significant terms included humoral and antimicrobial immune responses, response to bacterium, response to transforming growth factor beta, blood vessel morphogenesis, extracellular matrix organization, and epithelial cell proliferation (**Figure 3F**). Combining with the heatmap analysis, the specific transcriptional phenotypic evolution at each stage was further clarified (**Figure 3B**): the Control group showed expression of genes related to normal physiological functions; the 1M group presented transcriptional characteristics of early damage and stress states; while the 5M group showed higher expression of genes related to late fibrosis remodeling, matrix degradation, and immune infiltration.

### 3.4 Macrophage–fibroblast interaction network and SPP1-centered signaling

To explore cell–cell communication within the microenvironment, we used CellChat to construct predicted interaction networks at different stages. Overall predicted communication increased at 5M, especially for signaling involving macrophages (**Figure 4C**). In the macrophage-to-fibroblast analysis, SPP1 was among the strongest 5M pathways, with Spp1–(Itga8+Itgb1) and Spp1–(Itga9+Itgb1) among the detected ligand–receptor pairs (**Figure 4A**). In the separate pathway-wide analysis across all 5M cell populations, Spp1–Cd44 had the highest relative contribution (**Figure 4B**). SPP1 communication was minimal in Control and 1M but prominent at 5M (**Figure 4D**). At the single-cell level, normalized macrophage Spp1 expression increased across Control, 1M, and 5M, whereas normalized fibroblast Cd44 expression was lower at 1M and higher at 5M than in the other conditions (**Figure 4E**).

### 3.5 Wound healing and histological evaluation of boundary-spanning and fully irradiated wounds

This experiment consisted of four groups: NC group, H group, W group, and V group (**Figure 5A**). Morphologically, the NC group and the H group showed rapid wound contraction on the 14th day, with almost complete healing, while the W group exhibited marked repair delay and a larger residual wound (**Figures 5B–D**). Quantitative analysis revealed no significant difference in the healing rate between the NC group and the H group, but the healing rate of the W group was significantly lower compared to both (P < 0.0001, **Figure 5E**).

The qPCR results of mouse wound tissues showed that the expression of CCL2 and SERPINE1 was upregulated in the W group, while the expression of PDGFB and AREG was downregulated. The overall trend in the H group was a decrease, with only a slight increase in CCL2 (**Figure 5H**). From a histological perspective, the H group displayed dense granulation tissue rich in fibroblasts, while the wounds in the W group were thinner and less dense (**Figures 6A–D**).

Masson staining and COL1A1 immunohistochemistry results showed that the collagen fibers in the W group were sparse and loose, while the collagen bundles in the H group were more compact (**Figure 6E**). The expression of COL1A1 in fully irradiated wounds (R5W, R9W, R13W) was significantly lower than that in boundary-spanning wounds (R1H, R5H, R9H, R13H) (**Figures 6E–G**).

### 3.6 Immune cell infiltration, angiogenesis, and fibrotic marker expression

The CD68 immunostaining showed that a large number of macrophages were present in both the H group and the W group (**Figure 7A**). In the W group, the macrophages were more densely distributed around the wound than in the center. Time-series analysis revealed that the peak of macrophages in the periphery occurred early (H and W_P, R1), followed by a decline, while the macrophages in the center (W_C) increased over time (**Figure 7B**).

The CD31 immunohistochemistry results showed that the vascular density was highest in the normal control group (NC), followed by the boundary-spanning H group, and lowest in the fully irradiated W group (**Figures 7C, D**). Ki67 staining indicated that cell proliferation in the NC and W groups was higher than that in the H group. The W group reached its peak on the 9th day, while the H group reached its peak on the 1st day (**Figures 7D, E**). TGF-β and α-SMA staining levels were lower in the W group, whereas Smad2/3 staining was higher. TGF-β and α-SMA staining levels were higher in the H group, whereas Smad2/3 staining was lower (**Figures 7D, F–H**).

The Western blot analysis of mouse tissues showed that the expression order of TGF-β was NC > H > W. For α-SMA, the expression in H was slightly higher than that in NC, and the expression in W was the lowest. The expression of CD68 was highest in H, followed by W and NC, and their expression levels were similar (**Figures 5F, G**). Full uncropped western blot images are provided in **Supplementary Figure 1**.

## 4 Discussion

This study, based on secondary analysis of public lung scRNA-seq data (11) and an independent boundary-spanning skin wound model, examined temporal remodeling of the lung microenvironment and regional variation in short-term skin repair, and focused on revealing the phased transformation of fibroblast functional states and their potential communication signals. Overall, the two systems provide complementary observations rather than a single validated disease timeline.

The whole-cell map shows that in the early stage of the disease (1M), the proportions of major structural cells (fibroblasts, epithelial cells, endothelial cells) remained generally stable, while in the late stage (5M), these structural cell proportions decreased, and at the same time, the proportions of immune cells and adipocytes increased (**Figure 1B**). This phenomenon suggests that in the late stage of RIF, the microenvironment may shift from a “local tissue injury response” to an “immune-enriched microenvironmental remodeling” state (32), which is also consistent with the characterization of fibroblast gene enrichment (**Figures 3B–F**).

A notable finding is that ECM-related transcription and myofibroblast function show temporal decoupling. After Benjamini–Hochberg correction, ECM scores were higher at 1M and 5M than Control and higher at 5M than 1M, whereas the myofibroblast function score was lower at 5M than 1M but did not differ between 1M and Control (**Figures 1E, G**). The late-stage increase in ECM-related transcription was not accompanied by a parallel increase in the TGF-β signaling score, which did not differ significantly among time points (**Figure 1D**). This pattern is compatible with temporal asynchrony among classical TGF-β signaling, the myofibroblast contractile program, and ECM remodeling; it does not establish a three-way mechanistic decoupling. Together with the increased proportion of ECM fibroblasts at 5M (**Figure 2**), the findings are compatible with a transition toward ECM-dominant remodeling (32, 33).

In fact, the phenomenon that “TGF-β signaling and ECM deposition are not strictly synchronous in the fibrosis process” has been reported in previous studies (34). Although the classic view holds that TGF-β is the core factor driving the activation of myofibroblasts and excessive ECM deposition (35), more and more studies suggest that ECM remodeling in the later stage of fibrosis may enter a partially TGF-β-independent maintenance state (32, 36). This temporal asynchrony may help explain, but does not establish, why anti-TGF-β therapy can have limited efficacy in late-stage fibrosis (35, 37).

The inferred temporal sequence further supports the above observation. Several Slingshot branches pass from quiescent toward ECM-enriched fibroblast states before extending to other endpoints (**Figure 3A**). This recurrent, model-based pattern suggests that ECM fibroblasts may represent a potential intermediate or hub state, but it does not establish a required biological sequence or direct lineage relationship (37, 38).

Cell communication analysis further revealed potential stage-associated signals. The predicted intensity of macrophage communication increased over time, and 5M exhibited an SPP1 signaling network. In the pathway-wide analysis, Spp1–Cd44 had the highest relative contribution (**Figure 4B**), whereas the macrophage-to-fibroblast analysis identified SPP1–integrin interactions (**Figure 4A**). SPP1 (osteopontin) is considered an important bridging molecule between inflammation and fibrosis (39, 40). Macrophage Spp1 increased across stages, whereas fibroblast Cd44 showed more modest condition-associated variation (**Figure 4E**). These computational findings nominate SPP1-centered signaling as a candidate feature of 5M remodeling but do not establish causality or a macrophage-to-fibroblast SPP1–CD44 mechanism.

Using the independent skin model, this study examined repair at the boundary between irradiated and non-irradiated tissue and observed that a rapid matrix-rich response occurred when the wound crossed the irradiation boundary, whereas wounds located completely within the irradiated area showed healing impairment. This phenotype was observed within 14 days and therefore represents an accelerated acute/subacute matrix-rich response (**Figures 5**, **6**).

A possible explanation for the NC–H similarity is that the non-irradiated portion of a boundary-spanning wound contributed viable cells or paracrine support; this remains speculative because edge dosimetry and mechanistic tracing were not performed. By analogy with previous reports of radiation bystander effects (41, 42), this result raises the possibility of a cross-regional tissue response; however, a bystander mechanism was not directly tested in this model. The spatial differences between the two types of wounds show an opposite trend with the healing outcomes: boundary-spanning H wounds exhibit an accelerated phenotype rich in matrix, whereas fully irradiated W wounds show impaired repair function. This contrast indicates that the irradiation boundary microenvironment may affect the balance between wound healing and matrix remodeling.

In this model, TGF-β/SMAD activity, myofibroblast markers, and collagen deposition were not strictly synchronous across wound configurations and irradiation-to-wounding intervals (**Figures 6**, **7**). At the same time, the differences in vascular density and cell proliferation ability between wound configurations further indicated that the tissue microenvironment is reshaped under spatial heterogeneity and enters different repair trajectories (**Figures 7C–H**). The higher Ki67 positivity in W than H despite slower closure may reflect a compensatory but ineffective proliferative response and requires cell-type-specific investigation (43). Because TGF-β1, Smad2/3, and α-SMA differed in direction and were quantified by staining intensity/area without a formal localization model, these findings were not interpreted as pathway inactivation. The qPCR results suggest that while inflammatory and fibrotic signals were enhanced, tissue repair ability was inhibited. This expression pattern is conceptually complementary to the lung single-cell results, but cross-system differences preclude direct validation (**Figure 5H**). Notably, although Masson staining was weak in R1W, COL1A1 expression increased, suggesting that molecular matrix changes may precede mature collagen formation in this skin-wound setting (**Figure 6E**) (44).

Further immunohistochemical analysis showed that macrophage abundance varied across regions and time, with greater early peripheral accumulation and later central accumulation in some fully irradiated wounds (**Figures 7A, B**). This regional pattern broadly parallels the continuous increase in the proportion of macrophages and the predicted SPP1 communication pathway (39) in the lung single-cell transcriptomic dataset. However, SPP1 protein was not measured in the skin model, so this cross-system comparison remains an analogy rather than validation.

In summary, secondary analysis of public single-cell RNA-sequencing data indicated a temporal decoupling between transcriptional activity associated with ECM and myofibroblast function, along with predicted SPP1-centered intercellular communication and a shift toward ECM remodeling dominance at 5 months. The independent boundary-spanning wound model showed rapid tissue responses with marked spatial heterogeneity and indicated that TGF-β/SMAD-related markers, myofibroblast markers, and collagen deposition were not strictly synchronized. Taken together, these complementary but non-equivalent systems highlight the temporal and spatial heterogeneity of radiation-induced fibrosis, providing a foundation for future mechanistic studies.

## 5 Limitations

Several limitations exist in this study and should be taken into account. Firstly, the sample size of the mouse model used in the experiment was limited, and the wound healing and fibrotic response were only observed at short-term time points. The H-group phenotype observed at day 14 represents a rapid acute/subacute matrix-rich response rather than established chronic or late-stage radiation fibrosis. In the future, the number of animals can be increased and the observation period can be extended to verify the universality and long-term effects of the phenomenon. At the same time, this study did not specifically assess sex differences, so potential sex-related variation may not have been detected; future experiments can use sex-stratified designs to explore differential responses. Secondly, the depth of mechanism exploration is still limited, and only the TGF-β/Smad signaling is involved. Other possible pathways (such as IL-6, ROS, etc.) need to be further analyzed (45). In addition, the mouse model and the clinical radiotherapy environment have differences. The experimental 12 Gy single-dose exposure also differs from fractionated clinical radiotherapy. In the future, more similar clinical models or patient samples can be combined for verification. In terms of experimental methods, this study mainly relied on IHC, qPCR, and Western blot to detect key indicators, and did not conduct high-throughput genomics or single-cell analysis, which may have missed some potential mechanism information. The SPP1-centered communication findings are based on CellChat prediction and lack functional perturbation. The lung scRNA-seq and skin-wound models differ in tissue, dose, injury context, and time scale, limiting direct comparison. In addition, several single-cell comparisons used cells rather than biological samples as the unit of analysis and may therefore be affected by pseudoreplication; because the 1M and 5M groups each contained only two biological samples, the resulting P values should be interpreted as exploratory cell-level evidence rather than biological-replicate-level inference. Because the Western blot and qPCR assays used pooled group-level tissues, these molecular readouts were interpreted descriptively rather than used for animal-level inferential statistics. Wound-edge dosimetry, shielding scatter, formal blinding, and independent biological replication for the pooled Western blots were not available.

## Data Availability

The original contributions presented in the study are included in the article/**Supplementary Material**. Further inquiries can be directed to the corresponding author.
