# Supplementary material for: Single-cell transcriptomics and a boundary wound model reveal temporal decoupling and microenvironmental heterogeneity in radiation-induced fibrosis
Source: Front Oncol. 2026 Aug 20;16:1871514. doi: 10.3389/fonc.2026.1871514 (PMC13537982; doi:10.3389/fonc.2026.1871514)

Supplementary Figure S1. Full uncropped western blot images corresponding to Fig. 4E.

Fig S1A Raw blot for GAPDH

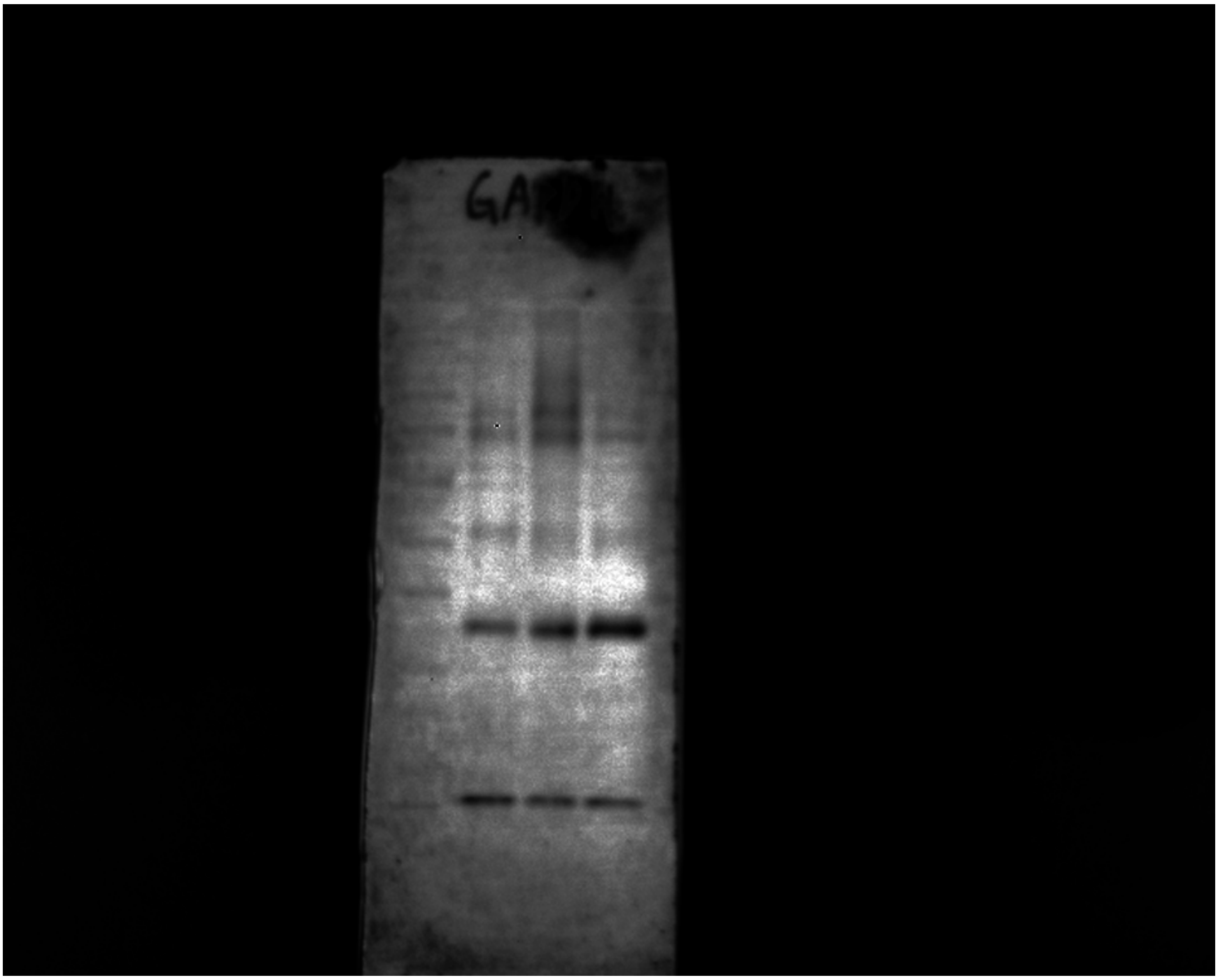

Fig S1B Raw blot for TGF-β

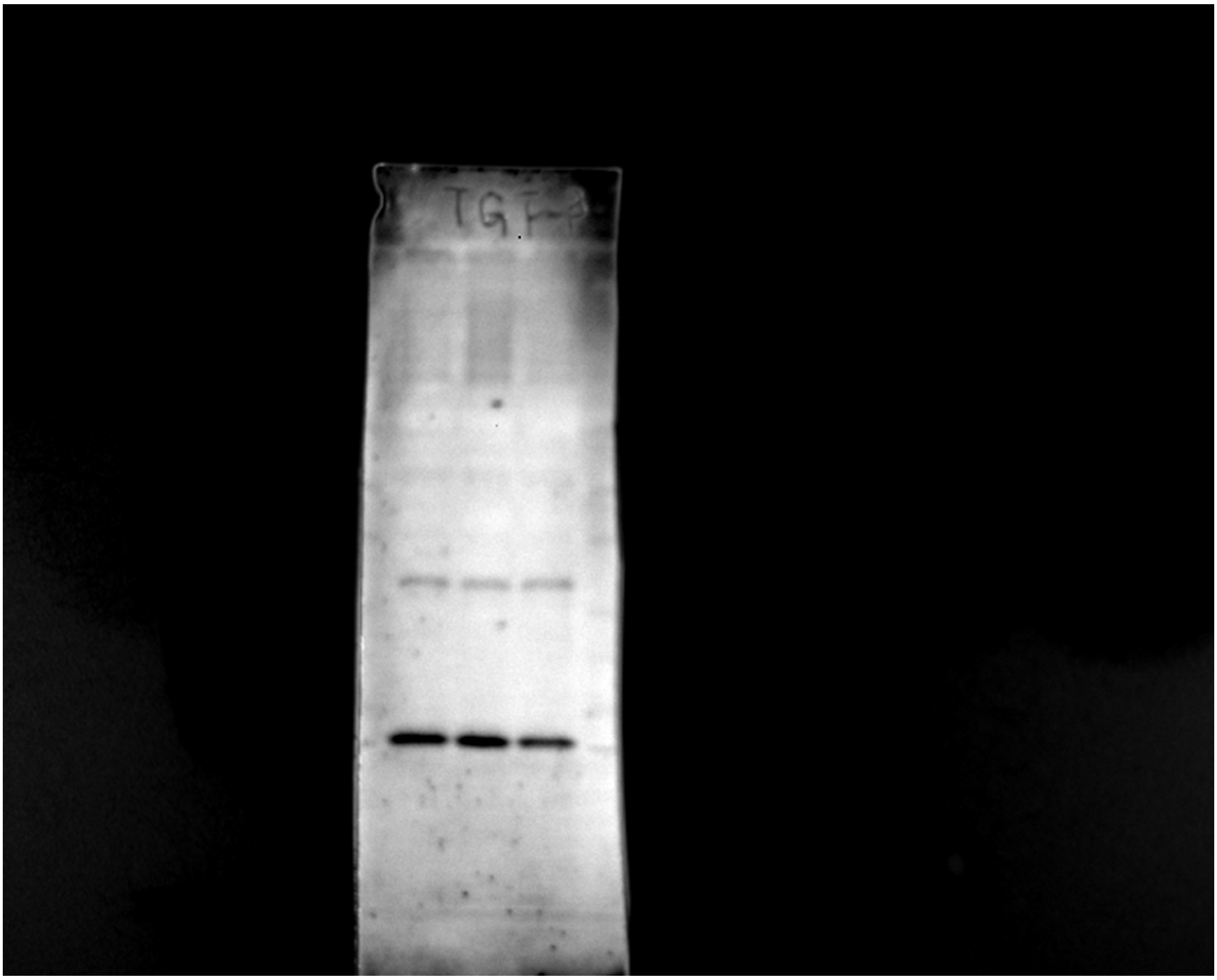

Fig S1C Raw blot for SMA

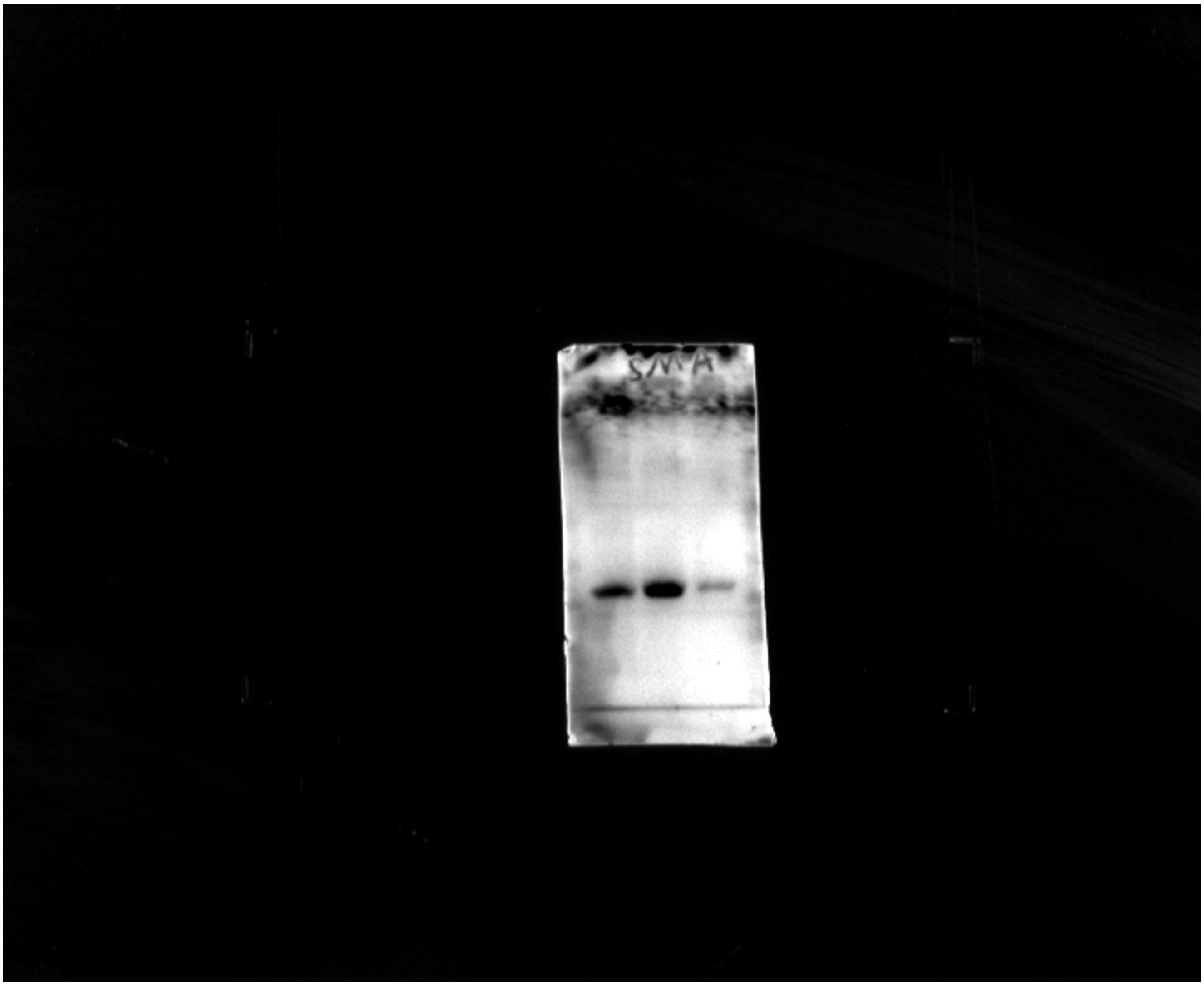

Fig S1D Raw blot for CD68

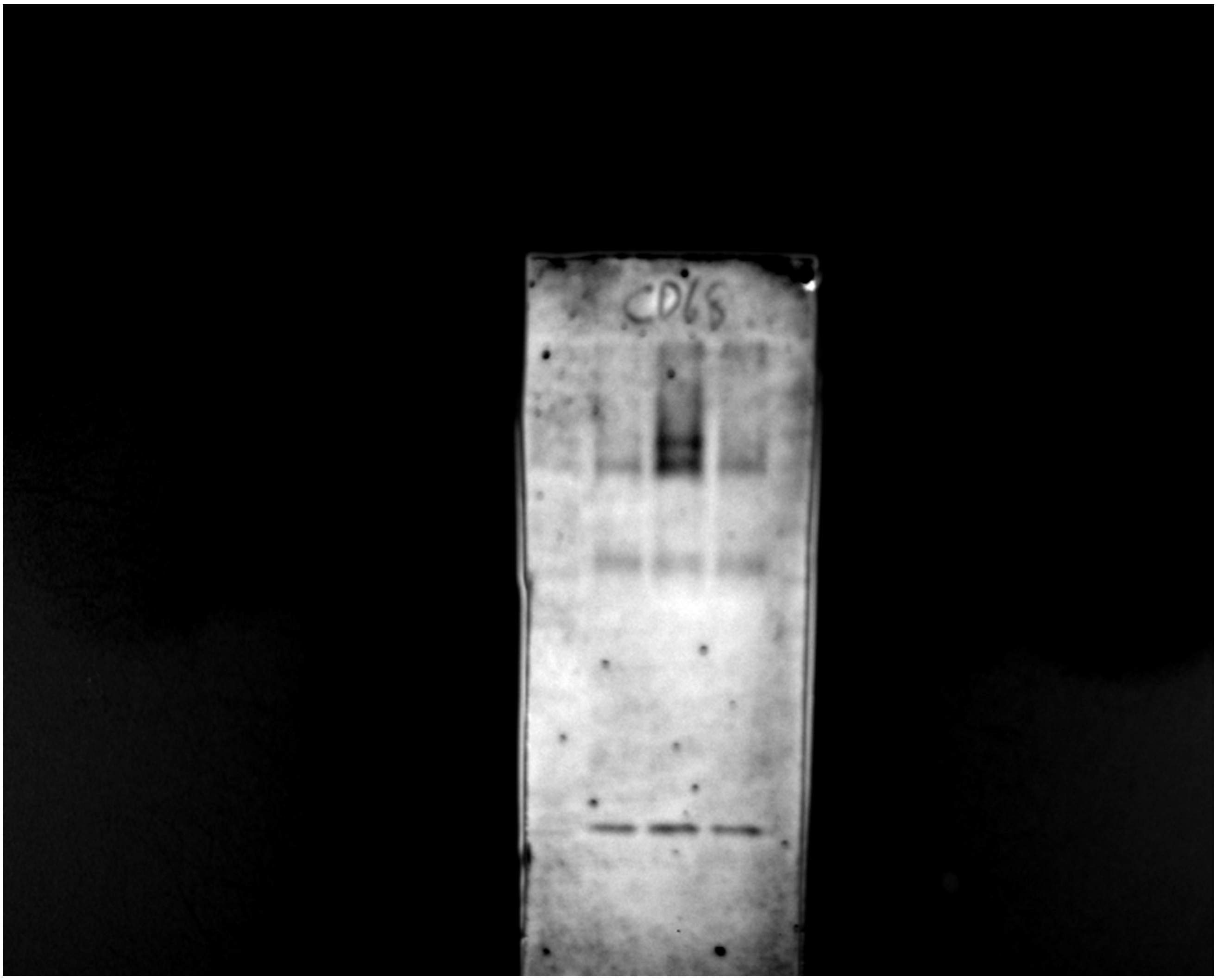

Fig S1E Annotated version of all blots. A single SDS–PAGE gel was used to transfer 3-4 membranes, and the molecular weight marker lane was used as the dividing line when cutting the membranes after transfer. Because the sample wells were relatively narrow and the marker lane was split during cutting, the marker bands appear very faint in the final blots. To make the marker bands more visible, the images were adjusted for brightness and contrast. Nevertheless, all marker bands were loaded according to the manufacturer's instructions, and the key marker positions adjacent to each target protein remain identifiable. Faint marker bands have been indicated in the figure.

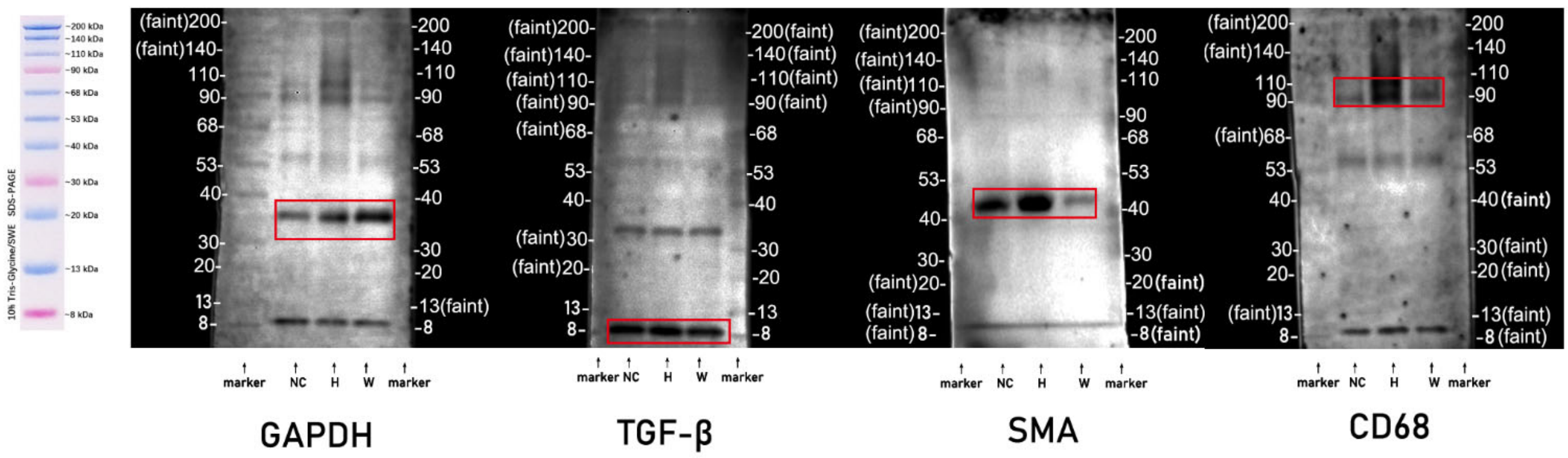

Supplement: Supplementary file 1 [file DataSheet1.pdf]
