# Supplementary material for: Single-cell transcriptomics and a boundary wound model reveal temporal decoupling and microenvironmental heterogeneity in radiation-induced fibrosis
Source: Front Oncol. 2026 Aug 20;16:1871514. doi: 10.3389/fonc.2026.1871514 (PMC13537982; doi:10.3389/fonc.2026.1871514)
Supplement: Supplementary file 2 [file DataSheet2.pdf]

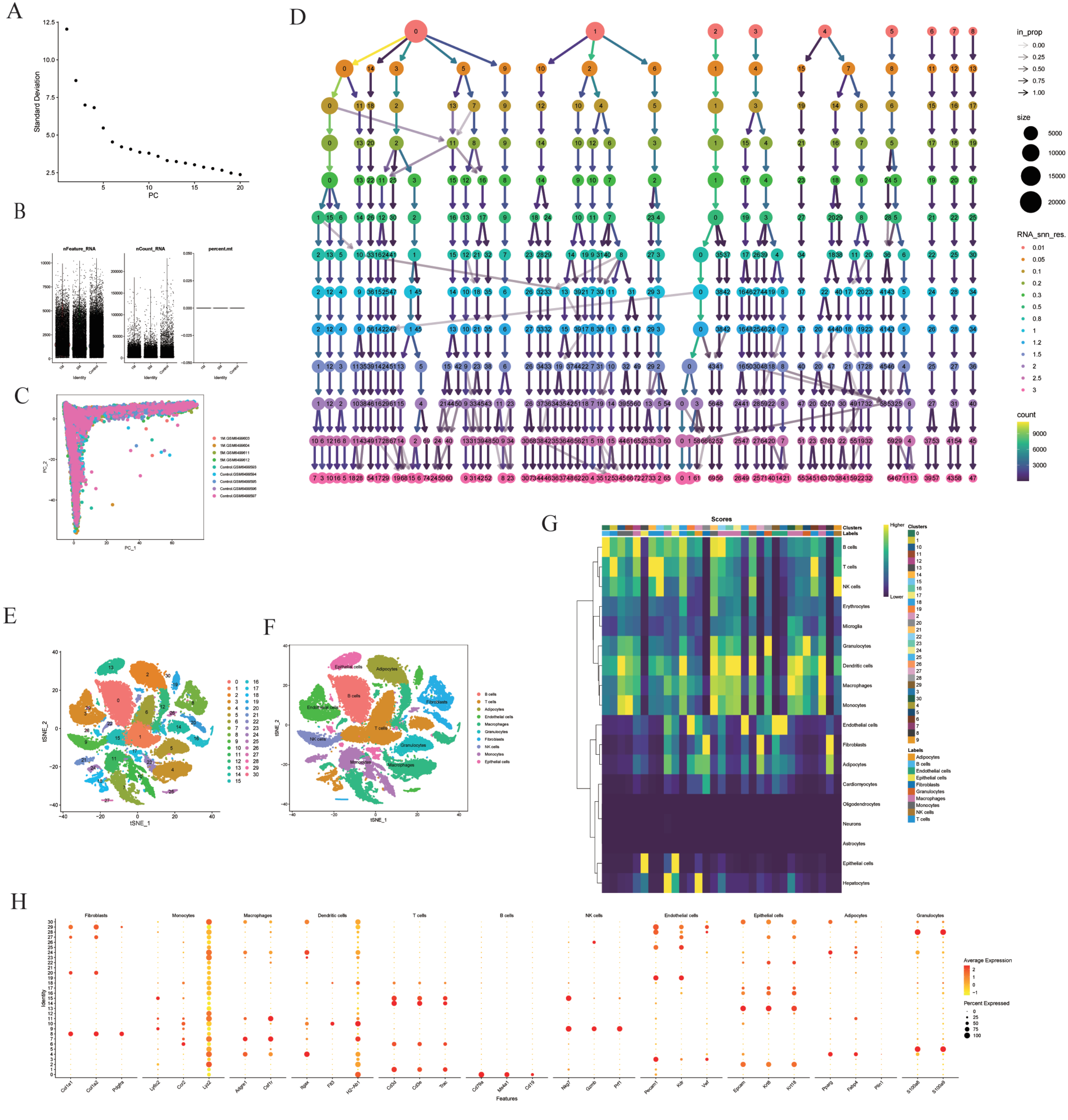

A
Standard Deviation
PC
B
nFeature_RNA
nCount_RNA
percent.mt
Identity
C
PC_1
PC_2
1M.GSM6499603
1M.GSM6499604
5M.GSM6499611
5M.GSM6499612
Control.GSM6499593
Control.GSM6499594
Control.GSM6499595
Control.GSM6499596
Control.GSM6499597
D
in_prop
size
RNA_snn_res.
count
E
tSNE_1
tSNE_2
F
B cells
T cells
Adipocytes
Endothelial cells
Macrophages
Granulocytes
Fibroblasts
NK cells
Monocytes
Epithelial cells
G
Scores
Clusters
Labels
Higher
Lower
B cells
T cells
NK cells
Erythrocytes
Microglia
Granulocytes
Dendritic cells
Macrophages
Monocytes
Endothelial cells
Fibroblasts
Adipocytes
Cardiomyocytes
Oligodendrocytes
Neurons
Astrocytes
Epithelial cells
Hepatocytes
H
Fibroblasts
Monocytes
Macrophages
Dendritic cells
T cells
B cells
NK cells
Endothelial cells
Epithelial cells
Adipocytes
Granulocytes
Identity
Features
Col1a1
Col1a2
Pdgfra
Ly6c2
Ccr2
Lyz2
Adgre1
Csf1r
Itgax
Flt3
H2-Ab1
Cd3d
Cd3e
Trac
Cd79a
Ms4a1
Cd19
Nkg7
Gzmb
Prf1
Pecam1
Kdr
Vwf
Epcam
Krt8
Krt18
Pparg
Fabp4
Plin1
S100a8
S100a9
Average Expression
Percent Expressed
