# Supplementary material for: Single-cell transcriptomics and a boundary wound model reveal temporal decoupling and microenvironmental heterogeneity in radiation-induced fibrosis
Source: Front Oncol. 2026 Aug 20;16:1871514. doi: 10.3389/fonc.2026.1871514 (PMC13537982; doi:10.3389/fonc.2026.1871514)
Supplement: Supplementary file 3 [file DataSheet3.pdf]

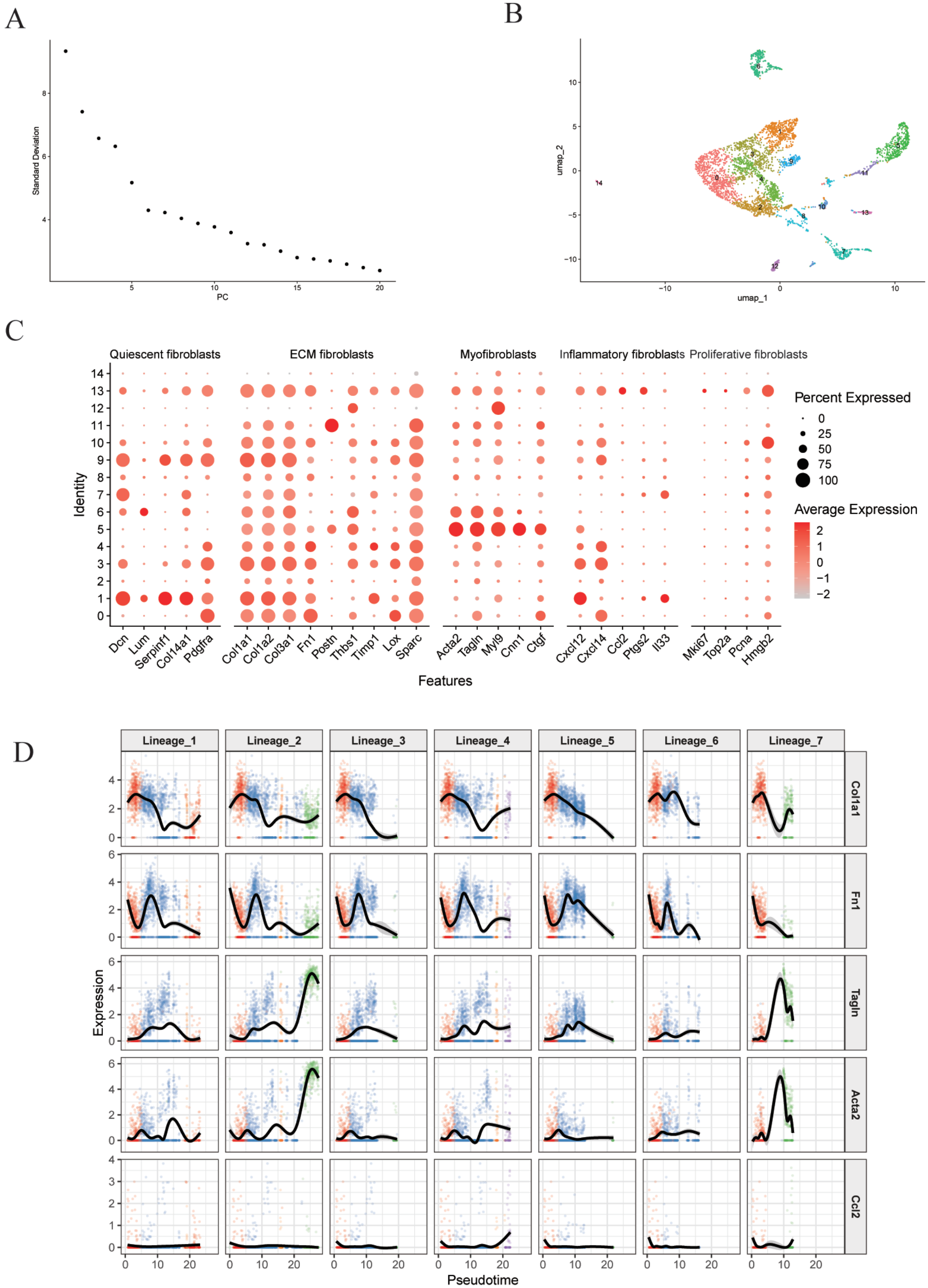
A
B
C
D
Standard Deviation
PC
umap_1
umap_2
Quiescent fibroblasts
ECM fibroblasts
Myofibroblasts
Inflammatory fibroblasts
Proliferative fibroblasts
Identity
Features
Dcn
Lum
Serpinf1
Col14a1
Pdgfra
Col1a1
Col1a2
Col3a1
Fn1
Postn
Thbs1
Timp1
Lox
Sparc
Acta2
Tagln
Myl9
Cnn1
Ctgf
Cxcl12
Cxcl14
Ccl2
Ptgs2
Il33
Mki67
Top2a
Pcna
Hmgb2
Percent Expressed
Average Expression
Lineage_1
Lineage_2
Lineage_3
Lineage_4
Lineage_5
Lineage_6
Lineage_7
Col1a1
Fn1
Tagln
Acta2
Ccl2
Expression
Pseudotime
