# Supplementary material for: Single-cell transcriptomics and a boundary wound model reveal temporal decoupling and microenvironmental heterogeneity in radiation-induced fibrosis
Source: Front Oncol. 2026 Aug 20;16:1871514. doi: 10.3389/fonc.2026.1871514 (PMC13537982; doi:10.3389/fonc.2026.1871514)
Supplement: Supplementary file 4 [file DataSheet4.pdf]

Table S1. Primer sequences used for quantitative PCR.

Mu-GAPDH-F	AGGTCGGTGTGAACGGATTTG
Mu-GAPDH-R	TGTAGACCATGTAGTTGAGGTCA
Mu-Ccl2-F	ACAAGAGGATCACCAGCAGC
Mu-Ccl2-R	GGACCCATTCCTTCTTGGGG
Mu-Pdgfb-F	GTGAACTTTGGAGCCTTGCAG
Mu-Pdgfb-R	TGCTCGGGTCATGTTCAAGT
Mu-Areg-F	TTGCTGCTGGTCTTAGGCTC
Mu-Areg-R	TGGTCCCCAGAAAGCGATTC
Mu-Pdgfra-F	CCTCAGAGAGAATCGGCCC
Mu-Pdgfra-R	TGGTGGGAGGTCCCCATAG
Mu-Serpine1-F	CCGATGGGCTCGAGTATGAC
Mu-Serpine1-R	TTCTCAAAGGGTGCAGCGAT
